# A Class of Algorithms for Recovery of Continuous Relaxation Spectrum from Stress Relaxation Test Data Using Orthonormal Functions

**DOI:** 10.3390/polym15040958

**Published:** 2023-02-15

**Authors:** Anna Stankiewicz

**Affiliations:** Department of Technology Fundamentals, Faculty of Production Engineering, University of Life Sciences in Lublin, 20-612 Lublin, Poland; anna.m.stankiewicz@gmail.com

**Keywords:** viscoelasticity, relaxation spectrum, linear relaxation modulus, identification algorithm, orthonormal functions, Tikhonov regularization, singular value decomposition

## Abstract

The viscoelastic relaxation spectrum provides deep insights into the complex behavior of polymers. The spectrum is not directly measurable and must be recovered from oscillatory shear or relaxation stress data. The paper deals with the problem of recovery of the relaxation spectrum of linear viscoelastic materials from discrete-time noise-corrupted measurements of relaxation modulus obtained in the stress relaxation test. A class of robust algorithms of approximation of the continuous spectrum of relaxation frequencies by finite series of orthonormal functions is proposed. A quadratic identification index, which refers to the measured relaxation modulus, is adopted. Since the problem of relaxation spectrum identification is an ill-posed inverse problem, Tikhonov regularization combined with generalized cross-validation is used to guarantee the stability of the scheme. It is proved that the accuracy of the spectrum approximation depends both on measurement noises and the regularization parameter and on the proper selection of the basis functions. The series expansions using the Laguerre, Legendre, Hermite and Chebyshev functions were studied in this paper as examples. The numerical realization of the scheme by the singular value decomposition technique is discussed and the resulting computer algorithm is outlined. Numerical calculations on model data and relaxation spectrum of polydisperse polymer are presented. Analytical analysis and numerical studies proved that by choosing an appropriate model through selection of orthonormal basis functions from the proposed class of models and using a developed algorithm of least-square regularized identification, it is possible to determine the relaxation spectrum model for a wide class of viscoelastic materials. The model is smoothed and robust on measurement noises; small model approximation errors are obtained. The identification scheme can be easily implemented in available computing environments.

## 1. Introduction

Viscoelasticity denotes the joint property of elasticity and viscosity and, hence, describes materials with both fluid and solid properties at the same time. Viscoelastic relaxation or retardation spectra are commonly used to describe, analyze, compare and improve the mechanical properties of polymers [1,2,3,4,5]. The spectra are vital for constitutive models and for the insight into the properties of a viscoelastic material, since, from the relaxation or retardation spectrum, other material functions used to describe rheological properties of various polymers can be uniquely determined [6,7,8,9]. However, the spectra are not directly accessible by measurement. The relaxation and retardation spectra can be recovered from oscillatory shear data and from the time measurements of the relaxation modulus or creep compliance obtained in standard stress relaxation or retardation experiments [1,2,7]. Many different methods have been proposed during the last five decades for relaxation spectrum computation using data from dynamic modulus tests. Baumgaertel and Winter [10] applied a nonlinear regression for identification of discrete relaxation and retardation time spectra based on dynamic data, in which the number of relaxation times adjusts during the iterative calculations to avoid ill-posedness and to improve the model fit; regularization is not applied here. Honerkamp and Weese [11,12], for relaxation spectrum identification, combined nonlinear regression with Tikhonov regularization and proposed a specific viscoelastic model described by the two-mode log-normal function. Malkin [13] approximated a continuous relaxation spectrum using three constants: the maximum relaxation time, slope in the logarithmic scale and form factor. Malkin et al. [14] derived a method of continuous relaxation spectrum calculations using the Mellin integral transform. An approach proposed by Stadler and Bailly [15] is based on the relaxation spectrum approximation by a piecewise cubic Hermite spline. In turn, Davies and Goulding [16] approximated the relaxation spectrum by a sum of scaling kernel functions located at appropriately chosen points. The algorithm for the relaxation time spectrum approximation by power series was developed by Cho [17]. Anderssen et al. [1] proposed a derivative-based algorithm for continuous spectrum recovery, being also appropriate for the experimental situation where the oscillatory shear data are only available for a finite range of frequencies. These works, but also many others, using different models, approaches, algorithms and computational techniques, have opened new directions of research on discrete and continuous relaxation spectra identification based on dynamic moduli data, which are still being conducted [2,18,19,20,21].

However, a classical manner of studying viscoelasticity is also through a two-phase stress relaxation test, where time-dependent shear stress is studied for the step increase in strain [4,6,7]. There are only a few papers, e.g., [22,23,24,25,26,27,28], that deal with the spectrum determination from time measurements of the relaxation modulus; additionally, only some of them are addressed to polymers. Therefore, the computationally efficient algorithms to determine the relaxation spectrum applied to time measurements of the relaxation modulus are still desirable. The objective of the present paper was to develop a class of models and an identification algorithm for the continuous relaxation spectrum determination based on discrete-time measurements of the relaxation modulus, which, taking into account the ill-posedness of the original problem of the spectrum recovery, will provide: (a) good approximation of the relaxation spectrum and modulus; (b) smoothness of the spectrum fluctuations, even for noise-corrupted measurements; (c) noise robustness; (d) applicability to a wide range of viscoelastic materials due to the choice of respective model from the considered set of models; (e) ease of implementation of the models and identification algorithm in available computing packages. Thus, the goal of this work was the synthesis of the respective models and general identification scheme, and the analysis of their properties. Approximation errors, convergence, noise robustness, smoothness and the applicability ranges were studied analytically. Further, the numerical verification of the models and algorithm for exemplary theoretical relaxation spectrum, and their applicability to spectrum of real material, polydisperse polymer, was the purpose of this work.

The approach proposed is based on the approximation of the spectrum by a finite linear combination of the basis orthonormal functions. A quadratic identification index, related to the data of the relaxation modulus, is adopted as a measure of the model quality. As a result, the primary infinite dimensional dynamic inverse problem of the continuous relaxation spectrum identification is reduced to the static linear-quadratic programming task. Next, Tikhonov regularization is used to guarantee the well-posed solution. Thus, the approach proposed integrates the technique of an expansion of a function into a series in an orthogonal basis with the least-squares regularized identification [29].

It is demonstrated that due to the choice of appropriate special functions as the basis functions for the unknown relaxation spectrum model, the components in the relaxation modulus model are given by compact analytical or recursive formulas. The technique of expanding an unknown viscoelastic function into a series of orthogonal functions or polynomials has already been used to describe various rheological models of polymers, especially in the time domain. For example, Aleksandrov et al. [30] applied Laguerre polynomials to describe experimentally obtained polyethylene deformation in the creep under diffusion in a liquid environment. Cao et al. [31] used orthogonal expansion based on shifted Legendre polynomials to solve a fractional-order viscoelastic model of polymethyl methacrylate. Abbaszadeh and Dehghan [32] employed a new class of basis based upon the Legendre polynomials to solve a two-dimensional viscoelastic equation. Kim et al. [33] used Chebyshev polynomials for direct conversion of creep data to dynamic moduli.

The idea of using a series of orthogonal functions has also been used to approximate the relaxation spectrum. Lee et al. [34] used the Chebyshev polynomials of the first kind to approximate dynamic moduli data. Stankiewicz [27,28,35] applied orthogonal functions for relaxation spectrum recovery from the stress relaxation data, but these articles use a different definition of the relaxation modulus, according to which the modulus is directly given by the Laplace integral of the spectrum. In this paper, it is shown, for the dominant literature definition of the relaxation spectrum, that the application of the concept of expanding the unknown relaxation spectrum into a series of orthonormal basis functions combined with the least-squares regularized identification allows one to determine the smoothed model of the relaxation spectrum, robust on the measurement noises, with small approximation errors of the relaxation spectrum and modulus. The selection of appropriate orthonormal basis functions, the selection of their time-scale factor and the determination of the optimal regularization parameter using standard generalized cross-validation technique enables the application of the proposed approach to a wide class of viscoelastic materials.

## 2. Materials and Methods

### 2.1. Relaxation Spectrum

The uniaxial, nonaging and isothermal stress–strain equation for a linear viscoelastic material can be represented by a Boltzmann superposition integral [7]:

(1) $$\sigma \left(t\right)=\int_{-\infty }^{t}G\left(t-\lambda \right)\dot{\varepsilon }\left(\lambda \right)d\lambda ,$$

where $\sigma \left(t\right)$ and $\varepsilon \left(t\right)$ denote the stress and strain at the time *t* and $G\left(t\right)$ is the linear relaxation modulus. Modulus $G\left(t\right)$ is given by [1,7,36,37]:

(2) $$G\left(t\right)=\int_{0}^{\infty }\frac{H\left(\tau \right)}{\tau }e^{-t/\tau }d\tau ,$$

or equivalently by

(3) $$G\left(t\right)=\int_{0}^{\infty }\frac{H\left(v\right)}{v}e^{-tv}dv,$$

where $H\left(\tau \right)$ and $H\left(v\right)$ characterize the distributions of relaxation times τ and relaxation frequencies v, respectively. The continuous relaxation spectra $H\left(\tau \right)$ and $H\left(v\right)$, related by $H\left(v\right)=H\left(\frac{1}{v}\right)$, are generalizations of the discrete Maxwell spectrum [1,7] to a continuous function of the relaxation times τ and frequencies v. Although other definitions of the relaxation spectrum are used in the literature, for example, in [5,13,28,38], the definition introduced by Equation (2) dominates. The main symbols are summarized in Nomenclature, Appendix C. 

The problem of relaxation spectrum determination is the practical problem of re-constructing the solution of the Fredholm integral equation of the first kind (2) or (3) from discrete-time measured data. Time measurements of the relaxation modulus data are considered in this paper. This problem is known to be severely Hadamard *ill-posed* [39,40]. In particular, small changes in the measured relaxation modulus can lead to arbitrarily large changes in the determined relaxation spectrum. In remedy, some reduction of the set of admissible solutions or appropriate regularization of the original problem can be used. Here, both techniques are used simultaneously. 

### 2.2. Models

The modified spectrum is introduced:

(4) $$H^{M}\left(v\right)=\frac{H\left(v\right)}{v},$$

where the upper index of $H^{M}\left(v\right)$ means ‘modified’. Then, (3) can be rewritten as

(5) $$G\left(t\right)=\int_{0}^{\infty }H^{M}\left(v\right)e^{-tv}dv,$$

i.e., the modulus is directly the Laplace integral of the spectrum $H^{M}\left(v\right)$.

Assume that $H^{M}\left(v\right)\in L^{2}\left(0,\right.\left.\infty \right)$, where $L^{2}\left(0,\right.\left.\infty \right)$ is the space of real-valued square-integrable functions on the interval $\left(0,\right.\left.\infty \right)$. The respective sufficient conditions are given by Theorem 3 in [41]. Assume that the set of the linearly independent orthonormal functions $\left\{h_{0}\left(v\right),h_{1}\left(v\right),h_{2}\left(v\right),\ldots \right\}$ form a basis of the space $L^{2}\left(0,\right.\left.\infty \right)$. Thus, the modified relaxation spectrum can be expressed as

(6) $$H^{M}\left(v\right)=\sum_{k=0}^{\infty }g_{k}h_{k}\left(v\right),$$

where the Fourier coefficients are [42]

$$g_{k}=\int_{0}^{\infty }H^{M}\left(v\right)h_{k}\left(v\right)dv.$$

For practical reasons and in order to reduce the set of admissible solutions, it is convenient to replace the infinite summation in Equation (6) with a finite one of *K* terms, i.e., to approximate the relaxation spectrum $H^{M}\left(v\right)$ by a model of the form

(7) $$H_{K}^{M}\left(v\right)=\sum_{k=0}^{K-1}g_{k}h_{k}\left(v\right),$$

where the lower index of $H_{K}^{M}\left(v\right)$ is the number *K* of model summands. Then, using (5), the respective model of the relaxation modulus is described by:

(8) $$G_{K}\left(t\right)=\int_{0}^{\infty }H_{K}^{M}\left(v\right)e^{-tv}dv=\sum_{k=0}^{K-1}g_{k}\phi_{k}\left(t\right),$$

where the functions

(9) $$\phi_{k}\left(t\right)=\int_{0}^{\infty }h_{k}\left(v\right)e^{-tv}dv.$$

Note, that function $\phi_{k}\left(t\right)$ is Laplace transform of $h_{k}\left(v\right)$ for real argument *t*, i.e.,

$$L\left[h_{k}\left(v\right)\right]=\phi_{k}\left(t\right),$$

with the notation $L\left[f\left(v\right)\right]$ used for Laplace transform. For basis functions, $h_{k}\left(v\right)$ applied in the developed algorithms, the components $\phi_{k}\left(t\right)$ of the relaxation modulus model $G_{K}\left(t\right)$ are given by analytical or recursive formulas. This avoids quadrature errors occurring in the numerical calculation of the integrals (9). The following special functions are considered as basis functions: Laguerre, Legendre, Chebyshev and Hermite. The respective basis functions $h_{k}\left(v\right)$ and $\phi_{k}\left(t\right)$ are described in Section 3.3, Section 3.4, Section 3.5 and Section 3.6. All basis functions depend on one parameter—time-scaling factor α.

### 2.3. Identification Problem

Identification consists of selecting, within the given class of models defined by (7), (8) such a model, which ensures the best fit to the measurement results. Suppose a certain identification experiment (stress relaxation test [4,6,7]) performed on the specimen of the material under investigation resulted in a set of measurements of the relaxation modulus $\left\{\overset{-}{G}\left(t_{i}\right)=G\left(t_{i}\right)+z\left(t_{i}\right)\right\}$ at the sampling instants $t_{i}\geq 0$, i=1,…,N, where $z\left(t_{i}\right)$ is additive measurement noise. It is assumed that the number of measurements N≥K. As a measure of model (8) accuracy, the square index is taken

(10) $$Q_{N}\left(g_{K}\right)=\sum_{i=1}^{N}{\left[\overset{-}{G}\left(t_{i}\right)-G_{K}\left(t_{i}\right)\right]}^{2},$$

where $g_{K}={\left[\begin{array}{ccc} g_{0} & \cdots & g_{K-1} \end{array}\right]}^{T}$ is a *K*-element vector of unknown coefficients of the models (7) and (8). Using the vector-matrix notation

(11) $$\Phi_{N,K}=\left[\begin{array}{ccc} \phi_{0}\left(t_{1}\right) & \cdots & \phi_{K-1}\left(t_{1}\right)\\ \vdots & \ddots & \vdots \\ \phi_{0}\left(t_{N}\right) & \cdots & \phi_{K-1}\left(t_{N}\right) \end{array}\right],\;{\overset{-}{G}}_{N}=\left[\begin{array}{c} \overset{-}{G}\left(t_{1}\right)\\ \vdots \\ \overset{-}{G}\left(t_{N}\right) \end{array}\right]$$

the identification index (10) can be rewritten in compact form as

(12) $$Q_{N}\left(g_{K}\right)={\left\|{\overset{-}{G}}_{N}-\Phi_{N,K}g_{K}\right\|}_{2}^{2},$$

where ${\left\|\cdot \right\|}_{2}$ denotes the square norm in the real Euclidean space $R^{N}$. Thus, the optimal identification of relaxation spectrum in the class of functions defined by (7) and (8) consists of solving, with respect to the model parameter $g_{K}$, the following least-squares problem:

(13) $$\underset{g_{K}\in R^{K}}{min}\;{\left\|{\overset{-}{G}}_{N}-\Phi_{N,K}g_{K}\right\|}_{2}^{2}.$$

The matrix $\Phi_{N,K}$ is usually ill-conditioned. Thus, the optimization problem (13) is still, like the original problem of solving Fredholm’s equation of the 1st kind (3), incorrectly posed in the sense of Hadamard. Consequently, the solution of (13) is not unique, i.e., there exist many optimal model parameters minimizing the identification index $Q_{N}\left(g_{K}\right)$ (12). However, even the normal (with the lowest Euclidean norm) solution of (13) is non-continuous and unbounded function of the measurement vector ${\overset{-}{G}}_{N}$. This means that when the data are noisy, even small changes in ${\overset{-}{G}}_{N}$ would lead to arbitrarily large artefacts in any optimal model parameter. To deal with the ill posedness, the Tikhonov regularization method is used, as presented below.

### 2.4. Regularization

Regularization aims to replace the ill-posed problem with a nearby well-posed problem. Tikhonov regularization [43] strives to stabilize the computation of the least-squares solution by minimizing a modified square functional of the form:

(14) $$\underset{g_{K}\in R^{K}}{min}\;{\left\|{\overset{-}{G}}_{N}-\Phi_{N,K}g_{K}\right\|}_{2}^{2}+\lambda {\left\|g_{K}\right\|}_{2}^{2},$$

where λ>0 is a regularization parameter. The above problem is well-posed; that is, the solution always exists, is unique, and continuously depends on both the matrix $\Phi_{N,K}$ and on the measurement data ${\overset{-}{G}}_{N}$. The parameter vector minimizing (14) is given by:

(15) $${\overset{-}{g}}_{K}^{\lambda }={\left(\Phi_{N,K}^{T}\Phi_{N,K}+\lambda I_{K,K}\right)}^{-1}\Phi_{N,K}^{T}{\overset{-}{G}}_{N},$$

where $I_{K,K}$ is K dimensional identity matrix. 

The choice of regularization parameter λ is crucial to identify the best model parameters. Here, we apply the generalized cross-validation GCV [39,44], which does not depend on *a priori* knowledge about the noise variance. The GCV technique relies on choosing, as a regularization parameter, λ*,* which minimizes the GCV functional defined by [44]

(16) $$V_{GCV}\left(\lambda \right)={\left\|\varrho \left(\lambda \right)\right\|}_{2}^{2}/{tr\left[\Xi \left(\lambda \right)\right]}^{2},$$

where the matrix

$$\Xi \left(\lambda \right)={I_{N,N}-\Phi_{N,K}\left(\Phi_{N,K}^{T}\Phi_{N,K}+\lambda I_{K,K}\right)}^{-1}\Phi_{N,K}^{T},$$

and

$$\varrho \left(\lambda \right)=\Xi \left(\lambda \right){\overset{-}{G}}_{N}={\overset{-}{G}}_{N}-\Phi_{N,K}{\overset{-}{g}}_{K}^{\lambda },$$

are the residual vector for the regularized solution (15); $tr\left[\Xi \left(\lambda \right)\right]$ denotes the trace of $\Xi \left(\lambda \right)$. The problem of choosing the optimal regularization parameter

(17) $$\lambda_{GCV}=min\left\{\lambda :\lambda =arg\;\underset{\lambda \geq 0}{\min}V_{GCV}\left(\lambda \right)\right\}.$$

has a unique solution and the resulting parameter ${\overset{-}{g}}_{K}^{\lambda_{GCV}}$ differs the least from the normal solution of problem (14) that we would obtain for the ideal (not noise corrupted) measurements of the relaxation modulus [44]. 

### 2.5. Algebraic Background

Formula (15) is generally unsuitable for computational purposes. The singular value decomposition (SVD, [45]) technique will be used. Let SVD of the N×K dimensional matrix $\Phi_{N,K}$ take the form [45]:

(18) $$\Phi_{N,K}=U\Sigma V^{T},$$

where $\Sigma =diag\left(\sigma_{1},\ldots ,\sigma_{r},0,\ldots ,0\right)\epsilon R^{N,K}$ is diagonal matrix containing the non-zero singular values $\sigma_{1},\ldots ,\sigma_{r}$ of the matrix $\Phi_{N,K}$ [45], matrices $V\in R^{K,K}$ and $U\in R^{N,N}$ are orthogonal and $r=rank\left(\Phi_{N,K}\right)<N$. Taking advantage of the diagonal structure of Σ and the matrices V and U orthogonality, it may be simply proved that the regularized optimal parameter ${\overset{-}{g}}_{K}^{\lambda }$ (15) is given by

(19) $${\overset{-}{g}}_{K}^{\lambda }={V\Lambda }_{\lambda }U^{T}{\overset{-}{G}}_{N},$$

where K×N diagonal matrix $\Lambda_{\lambda }$ is as follows:

(20) $$\Lambda_{\lambda }=diag\left(\sigma_{1}/\left(\sigma_{1}^{2}+\lambda \right),\ldots ,\sigma_{r}/\left(\sigma_{r}^{2}+\lambda \right),0,\ldots ,0\right).$$

Using SVD (18) and introducing N dimensional vector $Y=U^{T}{\overset{-}{G}}_{N}$, the GCV function (16) can be expressed by a convenient analytical formula

(21) $$V_{GCV}\left(\lambda \right)=\left[\sum_{i=1}^{r}\frac{\lambda^{2}y_{i}^{2}}{{\left(\sigma_{i}^{2}+\lambda \right)}^{2}}+\sum_{i=r+1}^{N}y_{i}^{2}\right]/{\left[N-r+\sum_{i=1}^{r}\frac{\lambda }{\left(\sigma_{i}^{2}+\lambda \right)}\right]}^{2},$$

as a function of the singular values $\sigma_{i}$ and elements $y_{i}$ of the vector Y. The function $V_{GCV}\left(\lambda \right)$ is differentiable for any λ; thus, an arbitrary gradient optimization method can be implemented to solve the GCV minimization task (17). 

## 3. Results and Discussion

In this section, a general scheme of the relaxation spectrum identification is given. The most important results for the evaluation of the effectiveness of the algorithm and models are presented, concerning the smoothing of the models, their accuracy for ideal and noisy measurements of the relaxation modulus and the linear convergence to the model that we would obtain for the noise-free measurements. Next, examples of orthonormal basis functions $h_{k}\left(v\right)$ and corresponding functions $\phi_{k}\left(t\right)$ are given.

### 3.1. Identification Algorithm

The determination of the model of relaxation spectrum involves the following steps.

1. Perform the experiment (stress relaxation test [4,7,46,47]) and record the measurements $\overset{-}{G}\left(t_{i}\right)$, i=1,…,N, of the relaxation modulus at times $t_{i}\geq 0$.
2. Choose the time-scaling factor α and the number K of model components comparing, for different values of α, a few first functions from the sequence $\left\{\phi_{k}\left(t\right)\right\}$ with the experiment results $\left\{\overset{-}{G}\left(t_{i}\right)\right\}$.
3. Compute the matrix $\Phi_{N,K}$ (11) and next determine SVD (18).
4. Determine GCV function $V_{GCV}\left(\lambda \right)$ (21), and next compute the optimal regularization parameter $\lambda_{GCV}$ minimizing $V_{GCV}\left(\lambda \right)$, i.e., solving the optimization task (17).
5. Compute the regularized solution ${\overset{-}{g}}_{K}^{\lambda }$ according to (19) for $\lambda =\lambda_{GCV}$.
6. For $\lambda =\lambda_{GCV}$, using ${\overset{-}{g}}_{K}^{\lambda }$ computed above, determine the modified spectrum of relaxation frequencies ${\overset{-}{H}}_{K}^{M}\left(v\right)$ according to:
  (22) $${\overset{-}{H}}_{K}^{M}\left(v\right)=\sum_{k=0}^{K-1}{\overset{-}{g}}_{k}^{\lambda }h_{k}\left(v\right).$$
7. Determine the spectrum of relaxation frequencies ${\overset{-}{H}}_{K}\left(v\right)$ according to
  (23) $${\overset{-}{H}}_{K}\left(v\right)={\overset{-}{H}}_{K}^{M}\left(v\right)v=\sum_{k=0}^{K-1}{\overset{-}{g}}_{k}^{\lambda }h_{k}\left(v\right)v.$$

#### Two Remarks

1. Only the SVD of the matrix, $\Phi_{N,K}$, of computational complexity $O\left(NK^{2}\right)$ [45] is a space- and time-consuming task of the scheme. However, the SVD must be computed only once and is accessible in the form of optimized numerical procedures in most commonly used computational packets.
2. The matrix $\Phi_{N,K}$ depends on the choice of the basis functions as well as the measurement points $t_{i}$; however, it does not depend on the relaxation modulus measurements $\overset{-}{G}\left(t_{i}\right)$. Thus, when the identification scheme is applied for successive samples of the same material, step 3 should not be repeated while the same time instants $t_{i}$ are kept and the same model parameters α and K are selected in step 2.

### 3.2. Analysis

In the context of the ill-posed inverse problem, for which the model quality index refers to the measured relaxation modulus but not directly to the unknown relaxation spectrum $H\left(v\right)$ and the modified spectrum $H^{M}\left(v\right)$ (4), we cannot estimate the error $\left\|{\overset{-}{H}}_{K}^{M}\left(v\right)-H^{M}\left(v\right)\right\|$ directly. As a reference point for the determined model ${\overset{-}{H}}_{K}^{M}\left(v\right)$ (22), we will consider several characteristics, as follows:

(a) The model of the relaxation spectrum that we would obtain on the basis of ideal (undisturbed) measurements of the relaxation modulus:

(24) $${\tilde{H}}_{K}^{M}\left(v\right)=\sum_{k=0}^{K-1}{\tilde{g}}_{k}^{\lambda }h_{k}\left(v\right),$$

where ${\tilde{g}}_{K}^{\lambda }$ is the vector of regularized solution of (14)

(25) $${\tilde{g}}_{K}^{\lambda }={\left(\Phi_{N,K}^{T}\Phi_{N,K}+\lambda I_{K,K}\right)}^{-1}\Phi_{N,K}^{T}G_{N},$$

for noise-free measurements of relaxation modulus $G_{N}={\left[\begin{array}{ccc} G\left(t_{1}\right) & \cdots & G\left(t_{N}\right) \end{array}\right]}^{T}$; c.f., Equation (11)

(b) The model of the relaxation spectrum that we obtain on the basis of the normal solution ${\overset{-}{g}}_{K}^{N}=\Phi_{N,K}^{†}{\overset{-}{G}}_{N}$ of the linear-quadratic problem (13) for noise measurements of the relaxation modulus:

(26) $${\overset{-}{H}}_{K}^{N}\left(v\right)=\sum_{k=0}^{K-1}{\overset{-}{g}}_{k}^{N}h_{k}\left(v\right),$$

where $\Phi_{N,K}^{†}$ is the Moore–Penrose pseudoinverse [48] of matrix $\Phi_{N,K}$, and ${\overset{-}{g}}_{k}^{N}$ is the elements of the vector ${\overset{-}{g}}_{K}^{N}$

(c) The model of the relaxation spectrum that we would obtain on the basis of the normal solution ${\tilde{g}}_{K}^{N}=\Phi_{N,K}^{†}G_{N}$ of the linear-quadratic problem (13) for noise-free measurements of the relaxation modulus:

(27) $${\tilde{H}}_{K}^{N}\left(v\right)=\sum_{k=0}^{K-1}{\tilde{g}}_{k}^{N}h_{k}\left(v\right).$$

Most of the results are formulated in terms of algebraic tools of the algorithm, i.e., SVD decomposition (18) of the matrix $\Phi_{N,K}$. Such an analysis enables a deeper insight into the properties of the algorithm and the resulting model. It shows not only the influence of the regularization parameter and measurement errors, but also the impact and significance of the selection of the basis functions, including their parameters and measurement points $t_{i}$, on which the singular values $\sigma_{i}$ of the matrix $\Phi_{N,K}$ depend. 

#### 3.2.1. Smoothness

The purpose of Tikhonov regularization relies on stabilization of the resulting vector ${\overset{-}{g}}_{K}^{\lambda }$. Due to the orthonormality of the basis functions $h_{k}\left(v\right)$ in the Hilbert space $L^{2}\left(0,\right.\left.\infty \right)$, for an arbitrary ${\overset{-}{H}}_{K}^{M}\left(v\right)$ of the form (22), the following equality holds

(28) $${\left\|{\overset{-}{H}}_{K}^{M}\left(v\right)\right\|}_{2}^{2}=\sum_{k=0}^{K-1}\sum_{j=0}^{K-1}{\overset{-}{g}}_{k}^{\lambda }{\overset{-}{g}}_{j}^{\lambda }\int_{0}^{\infty }h_{k}\left(v\right)h_{j}\left(v\right)dv=\sum_{k=0}^{K-1}{\left[{\overset{-}{g}}_{k}^{\lambda }\right]}^{2}={\left\|{\overset{-}{g}}_{K}^{\lambda }\right\|}_{2}^{2},$$

where ${\left\|\cdot \right\|}_{2}$ means the square norm, both in the real Euclidean space as well as in $L^{2}\left(0,\right.\left.\infty \right)$. Therefore, the smoothness of the optimal solution ${\overset{-}{g}}_{K}^{\lambda }$ of discrete problem (14) guarantees that the fluctuations in the respective spectrum of relaxation, in particular the resulting spectrum of relaxation ${\overset{-}{H}}_{K}^{M}\left(v\right)$ (22), are also bounded. In view of the above, due to orthonormality of the elements the basis system $\left\{h_{k}\left(v\right)\right\}$, the function ${\overset{-}{H}}_{K}^{M}\left(v\right)$ is the approximation of the real modified spectrum $H^{M}\left(v\right)$ in the class of functions $H_{K}^{M}\left(v\right)$ (7), optimal in the sense of the square identification index $Q_{N}\left(g_{K}\right)$ (10) of the bounded norm.

For any regularized ${\overset{-}{g}}_{K}^{\lambda }$ (19), bearing in mind the definition of the vector $Y=U^{T}{\overset{-}{G}}_{N}$ and orthogonality of V, we have ${\left\|{\overset{-}{g}}_{K}^{\lambda }\right\|}_{2}^{2}=Y^{T}\Lambda_{\lambda }^{T}V^{T}V\Lambda_{\lambda }Y=Y^{T}\Lambda_{\lambda }^{T}\Lambda_{\lambda }Y$. Thus, due to the diagonal structure of $\Lambda_{\lambda }$ (20) and based on (28), the model smoothing efficiency can be evaluated by the following relation:

(29) $${\left\|{\overset{-}{H}}_{K}^{M}\left(v\right)\right\|}_{2}^{2}=\sum_{i=1}^{r}\frac{\sigma_{i}^{2}y_{i}^{2}}{{\left(\sigma_{i}^{2}+\lambda \right)}^{2}}<\sum_{i=1}^{r}\frac{y_{i}^{2}}{\sigma_{i}^{2}}={\left\|{\overset{-}{H}}_{K}^{N}\left(v\right)\right\|}_{2}^{2},$$

which holds for an arbitrary regularization parameter λ>0, where the spectrum ${\overset{-}{H}}_{K}^{N}\left(v\right)$ is given by (26). The last equality in (29) holds, since for $\Phi_{N,K}$ (18), the Moore–Penrose pseudoinverse is $\Phi_{N,K}^{†}={V\Sigma }^{†}U^{T}$, where K×N matrix $\Sigma^{†}=diag\left(1/\sigma_{1},\ldots ,1/\sigma_{r},0,\ldots ,0\right)$. Keeping in mind (26) and (28), the above result can be derived directly from the following inequality, proved in [28]:

$${\left\|{\overset{-}{g}}_{K}^{\lambda }\right\|}_{2}^{2}=\sum_{i=1}^{r}\frac{{\overset{-}{y}}_{i}^{2}}{{\left({\overset{-}{\sigma }}_{i}+\lambda \right)}^{2}}<\sum_{i=1}^{r}\frac{{\overset{-}{y}}_{i}^{2}}{{\overset{-}{\sigma }}_{i}^{2}}={\left\|{\overset{-}{g}}_{K}^{N}\right\|}_{2}^{2},$$

where ${\overset{-}{\sigma }}_{1},\ldots ,{\overset{-}{\sigma }}_{r}$ are the non-zero singular values of the matrix $\Phi_{N,K}^{T}\Phi_{N,K}$ and ${\overset{-}{y}}_{i}$ are elements of the K dimensional vector $\overset{-}{Y}=V^{T}\Phi_{N,K}^{T}{\overset{-}{G}}_{N}=\Sigma^{T}Y$, since ${\overset{-}{\sigma }}_{i}=\sigma_{i}^{2}$ and ${\overset{-}{y}}_{i}=\sigma_{i}y_{i}$. 

The first equality in (29) illustrates the mechanism of stabilization. The following rule holds: the greater the regularization parameter λ is, the more highly bounded the fluctuations of the spectrum ${\overset{-}{H}}_{K}^{M}\left(v\right)$ are. Thus, due to orthogonality of the basis functions, the regularization parameter controls the smoothness, not only of the parameter ${\overset{-}{g}}_{K}^{\lambda }$ but also of the model ${\overset{-}{H}}_{K}^{M}\left(v\right)$. The non-zero singular values of the matrix $\Phi_{N,K}$ and the vector of measurement data ${\overset{-}{G}}_{N}$ also affect the smoothness of the spectrum model. 

#### 3.2.2. Convergence

Relaxation spectrum ${\overset{-}{H}}_{K}^{M}\left(v\right)$ (22) is only an approximation of that spectrum, which can be obtained in the class of models (7) by direct minimization (without regularization) of the quadratic index $Q_{N}\left(g_{K}\right)$ (12) for noise-free measurements, i.e., the approximation of the function ${\tilde{H}}_{K}^{N}\left(v\right)$ (27). Since

$${\overset{-}{g}}_{K}^{\lambda }-{\tilde{g}}_{K}^{N}=V{\left(\Lambda_{\lambda }-\Sigma^{†}\right)U}^{T}{\overset{-}{G}}_{N}+{V\Sigma }^{†}U^{T}z_{N},$$

where $z_{N}={\left[\begin{array}{ccc} z\left(t_{1}\right) & \cdots & z\left(t_{N}\right) \end{array}\right]}^{T}$ is vector of measurement noises, based on (28); the diagonal structure of $\left(\Lambda_{\lambda }-\Sigma^{†}\right)$ and using the Schwarz inequality [49], the following bound of the relaxation spectrum approximation error can be derived:

(30) $${\left\|{\overset{-}{H}}_{K}^{M}\left(v\right)-{\tilde{H}}_{K}^{N}\left(v\right)\right\|}_{2}={\left\|{\overset{-}{g}}_{K}^{\lambda }-{\tilde{g}}_{K}^{N}\right\|}_{2}\leq \sum_{i=1}^{r}\frac{\lambda \left|y_{i}\right|}{\sigma_{i}\left(\sigma_{i}^{2}+\lambda \right)}+\frac{1}{\sigma_{r}^{2}}{\left\|z_{N}\right\|}_{2}.$$

The inequality (30) yields that the accuracy of the spectrum approximation depends both on the measurement noises and the regularization parameter and on the singular values $\sigma_{1},\ldots ,\sigma_{r}$ of the matrix $\Phi_{N,K}$, which, in turn, depend on the selection of the basis orthogonal functions $h_{k}\left(v\right)$. Using (30), the regularized vector ${\overset{-}{g}}_{K}^{\lambda }$ converges to the noise-free normal solution ${\tilde{g}}_{K}^{N}$ linearly with respect to the norm ${\left\|z_{N}\right\|}_{2}$, as λ→0 and ${\left\|z_{N}\right\|}_{2}\to 0$, simultaneously. Therefore, the upper bound in (30) guarantees that the spectrum ${\overset{-}{H}}_{K}^{M}\left(v\right)$ tends to ${\tilde{H}}_{K}^{N}\left(v\right)$ in each point v, at which they are both continuous, as λ→0 and ${\left\|z_{N}\right\|}_{2}\to 0$, simultaneously. 

#### 3.2.3. Noise Robustness

The influence of disturbances in the measurements of the relaxation modulus on the regularized solution ${\overset{-}{g}}_{K}^{\lambda }$ was analyzed in detail in a two-part paper [27,50]. From Property 2 in [50], the following inequalities result:

$${\left\|{\overset{-}{H}}_{K}^{M}\left(v\right)-{\tilde{H}}_{K}^{M}\left(v\right)\right\|}_{2}={\left\|{\overset{-}{g}}_{K}^{\lambda }-{\tilde{g}}_{K}^{\lambda }\right\|}_{2}\leq \underset{1\leq i\leq r}{\max}\frac{\sigma_{i}}{\left(\sigma_{i}^{2}+\lambda \right)}{\left\|z_{N}\right\|}_{2}\leq \frac{\sigma_{1}}{\left(\sigma_{r}^{2}+\lambda \right)}{\left\|z_{N}\right\|}_{2}.$$

Thus, the regularized vector ${\overset{-}{g}}_{K}^{\lambda }$ converges to the noise-free regularized solution ${\tilde{g}}_{K}^{\lambda }$ (25), and the relaxation spectrum ${\overset{-}{H}}_{K}^{M}\left(v\right)$ tends to the noise-free spectrum ${\tilde{H}}_{K}^{M}\left(v\right)$ (24) in each point v, where they are both continuous, linearly with respect to the norm ${\left\|z_{N}\right\|}_{2}$, as ${\left\|z_{N}\right\|}_{2}\to 0$. From the above estimations, it is also evident that the accuracy of the spectrum approximation measured by ${\left\|{\overset{-}{H}}_{K}^{M}\left(v\right)-{\tilde{H}}_{K}^{M}\left(v\right)\right\|}_{2}$ depends both on the measurement noises and the regularization parameter λ and on the singular values of the matrix $\Phi_{N,K}$. 

### 3.3. Legendre Model

Let us assume a basis function

(31) $$h_{k}\left(v\right)=\sqrt{2\alpha \left(2k+1\right)}e^{-\alpha v}P_{k}\left(1-2e^{-2\alpha v}\right),k=0,1,2,\ldots ,$$

with the time-scaling factor α, where $P_{k}\left(x\right)$ is Legendre polynomials [51,52,53] defined by Rodrigue’s formula

$$P_{k}\left(x\right)=\frac{1}{2^{k}k!}\frac{d^{k}}{dx^{k}}{\left(x^{2}-1\right)}^{k},\;k=0,1,2,\ldots .$$

The polynomials $P_{k}\left(x\right)$ form a complete set of orthonormal basis in the interval $\left[-1,1\right]$ with the weight $\left(2k+1\right)/2$ [51,53]. Thus, using the substitution $x=1-2e^{-2\alpha v}$, it is easy to observe that the functions $h_{k}\left(v\right)$ defined by (31) form a complete orthonormal basis in $L^{2}\left(0,\right.\left.\infty \right)$ [49]. The relaxation modulus basis functions $\phi_{k}\left(t\right)$ (9) are as follows:

(32) $$\phi_{k}\left(t\right)=\sqrt{2\alpha \left(2k+1\right)}\frac{\prod_{i=0}^{k-1}\left[\left(2i+1\right)\alpha -t\right]}{\prod_{i=0}^{k}\left[\left(2i+1\right)\alpha +t\right]},k=0,1,2,\ldots ,$$

where the product $\prod_{i=0}^{p}x_{i}$ is equal to 1 when p<0. The proof by induction is presented in Appendix A.1. The above formula can be equivalently expressed in recurrent form as

$$\phi_{k+1}\left(t\right)=\phi_{k}\left(t\right)\frac{\sqrt{2k+3}\left[\left(2k+1\right)\alpha -t\right]}{\sqrt{2k+1}\left[\left(2k+3\right)\alpha +t\right]},k=0,1,2,\ldots ,$$

starting with

$$\phi_{0}\left(t\right)=\frac{\sqrt{2\alpha }}{\alpha +t}.$$

**Figure 1:**
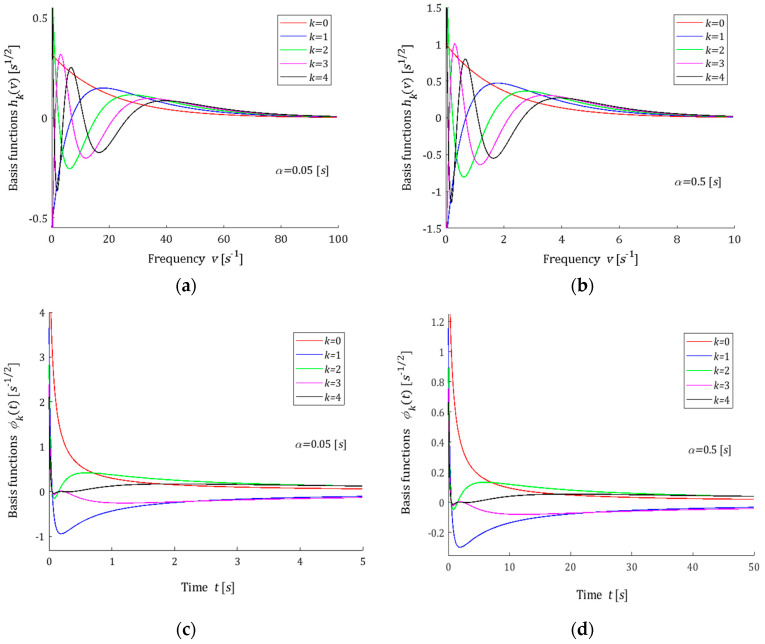
Basis functions $h_{k}\left(v\right)$ (31) and $\phi_{k}\left(t\right)$ (32) of the Legendre model for two time-scaling factors α: (**a**) $h_{k}\left(v\right)$, α=0.05[s]; (**b**) $h_{k}\left(v\right)$, α=0.5[s]; (**c**) $\phi_{k}\left(t\right)$, α=0.05[s]; (**d**) $\phi_{k}\left(t\right)$, α=0.5[s]; k=0,1,2,3,4.

Five first basis functions $h_{k}\left(v\right)$ are shown in Figure 1a,b for two different values of the time-scaling factor α. Figure 1c,d show the related $\phi_{k}\left(t\right)$ functions. From the last figure, it is seen that the basis functions for the relaxation modulus model are in good agreement with the real relaxation modulus obtained in the experiment. 

### 3.4. Laguerre Model 

The Laguerre polynomials can be defined via Rodrigue’s formula [51,54]:

(33) $$L_{k}\left(v\right)=\frac{e^{\alpha v}}{k!}\frac{d^{k}}{dv^{k}}\left(v^{k}e^{-\alpha v}\right),\;k=0,1,2,\ldots$$

where α>0 is a time-scaling factor [55]. The continuous Laguerre function is the product of the Laguerre polynomial and the square root of the exponential weight function $\alpha e^{-\alpha v}$ [56], i.e.,

(34) $$h_{k}\left(v\right)=\sqrt{\alpha }e^{-\alpha v/2}L_{k}\left(v\right),k=0,1,2,\ldots$$

The Laguerre functions form a complete orthonormal basis in $L^{2}\left(0,\right.\left.\infty \right)$ [51,56]. In Appendix A.2, the following formula is derived for the modulus basis functions:

(35) $$\phi_{k}\left(t\right)=\frac{\sqrt{\alpha }{\left(t-\frac{\alpha }{2}\right)}^{k}}{{\left(t+\frac{\alpha }{2}\right)}^{k+1}},\;k=0,1,2,\ldots .$$

The above formula is given by Wang and Cluett [55], but as there are several definitions of the Laguerre functions in the literature, and, as a result, several formulas of the Laplace transforms, for example, in [57], the derivation of (35) is given in Appendix A.2 to avoid doubts.

**Figure 2:**
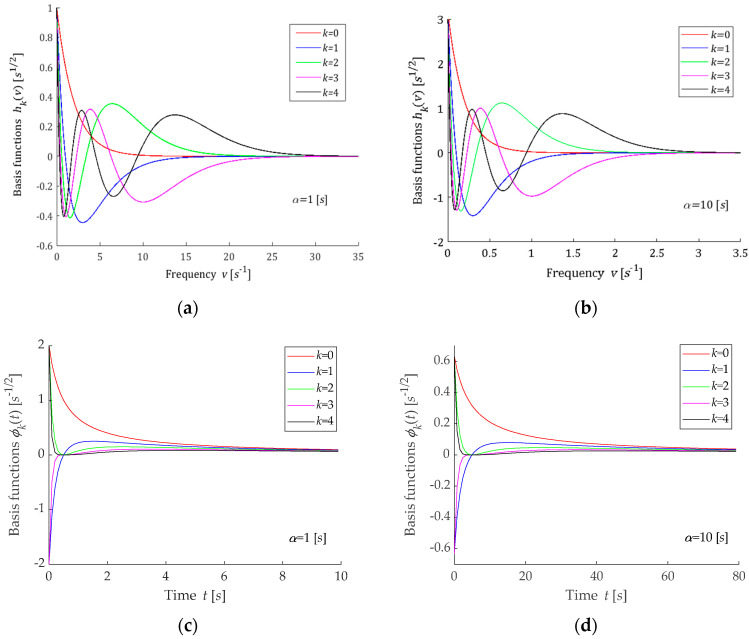
Basis functions $h_{k}\left(v\right)$ (34) and $\phi_{k}\left(t\right)$ (35) of the Laguerre model for two time-scaling factors: (**a**) $h_{k}\left(v\right)$, α=1[s]; (**b**) $h_{k}\left(v\right)$, α=10[s]; (**c**) $\phi_{k}\left(t\right)$, α=1[s]; (**d**) $\phi_{k}\left(t\right)$, α=10[s]; k=0,1,2,3,4.

A few first basis functions $h_{k}\left(v\right)$ are shown in Figure 2a,b for two different values of the time-scaling factor α; the corresponding functions $\phi_{k}\left(t\right)$ are plotted in Figure 2c,d. 

### 3.5. Chebyshev Model 

The Chebyshev polynomials of the first kind defined by the recursion relation [58,59]:

(36) $$T_{k}\left(x\right)=2xT_{k-1}\left(x\right)-T_{k-2}\left(x\right),\;k=2,3,\ldots ,$$

starting with

(37) $$T_{0}\left(x\right)=1,\;\;T_{1}\left(x\right)=x,$$

are orthogonal in the interval [−1,1] with the weight function ${\left(1-x^{2}\right)}^{-1/2}$ [58]. Specifically,

$$\int_{-1}^{1}\frac{T_{k}\left(x\right)T_{m}\left(x\right)}{\sqrt{1-x^{2}}}dx=\left\{\begin{array}{ll} 0 & k\neq m\\ \frac{\pi }{2} & k=m=1,2,\ldots \\ \pi & k=m=0 \end{array}\right.$$

**Figure 3:**
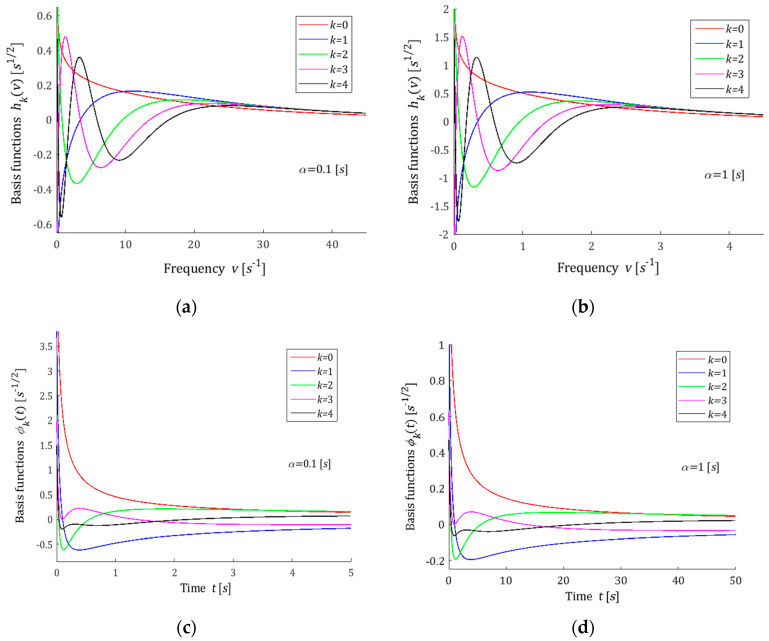
Basis functions $h_{k}\left(v\right)$ (38), (39) and $\phi_{k}\left(t\right)$ (40)–(43) of the Chebyshev model for two time-scaling factors: (**a**) $h_{k}\left(v\right)$, α=0.1[s]; (**b**) $h_{k}\left(v\right)$, α=1[s]; (**c**) $\phi_{k}\left(t\right)$, α=0.1[s]; (**d**) $\phi_{k}\left(t\right)$, α=1[s]; k=0,1,2,3,4.

Thus, using the substitution $x=\left(1-2e^{-2\alpha v}\right)$, it is easy to demonstrate that the set of functions

(38) $$h_{k}\left(v\right)=2\sqrt{\frac{\alpha }{\pi }}{\left(e^{2\alpha v}-1\right)}^{-1/4}T_{k}\left(1-2e^{-2\alpha v}\right),\;k=1,2,\ldots ,$$

with the first function defined as

(39) $$h_{0}\left(v\right)=\sqrt{\frac{2\alpha }{\pi }}{\left(e^{2\alpha v}-1\right)}^{-1/4},$$

form an orthonormal basis in the space $L^{2}\left(0,\right.\left.\infty \right)$. Here, as previously, α is a positive time-scaling factor. The relaxation modulus basis functions $\phi_{k}\left(t\right)$ (9) are described by a useful recursive formula

(40) $$\phi_{k}\left(t\right)=2\phi_{k-1}\left(t\right)-\phi_{k-2}\left(t\right)-4\phi_{k-1}\left(t+2\alpha \right),\;k=3,4,\ldots ,$$

and for k=0,1,2 are given by:

(41) $$\phi_{0}\left(t\right)=\frac{1}{\sqrt{2\pi \alpha }}\frac{\Gamma \left(\frac{3}{4}\right)\Gamma \left(\frac{t}{2\alpha }+\frac{1}{4}\right)}{\Gamma \left(\frac{t}{2\alpha }+1\right)},$$

(42) $$\phi_{1}\left(t\right)=\frac{\left(\alpha -t\right)}{2\alpha \sqrt{\pi \alpha }}\frac{\Gamma \left(\frac{3}{4}\right)\Gamma \left(\frac{t}{2\alpha }+\frac{1}{4}\right)}{\Gamma \left(\frac{t}{2\alpha }+2\right)},$$

(43) $$\phi_{2}\left(t\right)=\frac{\left(2\alpha^{2}+t^{2}-6\alpha t\right)}{4\alpha^{2}\sqrt{\pi \alpha }}\frac{\Gamma \left(\frac{3}{4}\right)\Gamma \left(\frac{t}{2\alpha }+\frac{1}{4}\right)}{\Gamma \left(\frac{t}{2\alpha }+3\right)},$$

where $\Gamma \left(n\right)$ is the Euler’s gamma function [60]. The proof is given in Appendix A.3, where two alternative formulas (A11) and (A12) for $\phi_{1}\left(t\right)$ and $\phi_{2}\left(t\right)$, respectively, are also derived. A few first basis functions $h_{k}\left(v\right)$ are shown in Figure 3a,b for two values of the factor α; the corresponding functions $\phi_{k}\left(t\right)$ are plotted in Figure 3c,d. An earlier version of the model was presented in the paper [61]. 

### 3.6. Hermite Model 

The Hermite functions defined as [52,62,63]

(44) $$h_{k}\left(v\right)=\frac{\sqrt{\alpha }}{\sqrt{2^{k}k!}\sqrt[{4}]{\pi }}e^{-{\left(\alpha v\right)}^{2}/2}H_{k}\left(\alpha v\right),\;k=0,1,\ldots ,$$

where α>0 is time-scaling factor and $H_{k}\left(x\right)$ are the Hermite polynomials, which satisfy recursion formula [52]:

(45) $$H_{k}\left(x\right)=2xH_{k-1}\left(x\right)-2\left(k-1\right)H_{k-2}\left(x\right),\;k=2,3,\ldots ,$$

with the initial

(46) $$H_{0}\left(x\right)=1,\;H_{1}\left(x\right)=2x,$$

constitute an orthonormal system in the space $L^{2}\left(-\infty ,\right.\left.\infty \right)$ [52,63]. The relaxation modulus basis functions $\phi_{k}\left(t\right)$ (9) are described by the recursive formula:

(47) $$\phi_{k}\left(t\right)=\frac{1}{\sqrt{2^{k-2}k!\alpha }\sqrt[{4}]{\pi }}H_{k-1}\left(0\right)+\sqrt{\frac{k-1}{k}}\phi_{k-2}\left(t\right)-\frac{\sqrt{2}}{\sqrt{k}\alpha }{t\phi }_{k-1}\left(t\right),k=2,3,\ldots$$

and for k=0,1 are given by

(48) $$\phi_{0}\left(t\right)=\frac{\sqrt[{4}]{\pi }}{\sqrt{2\alpha }}e^{t^{2}/\left(2\alpha^{2}\right)}erfc\left(\frac{t}{\sqrt{2}\alpha }\right),$$

and

(49) $$\phi_{1}\left(t\right)=\frac{\sqrt{2}}{\sqrt{\alpha }\sqrt[{4}]{\pi }}-\frac{\sqrt{2}}{\alpha }t\phi_{0}\left(t\right),$$

where the complementary error function $erfc\left(x\right)$ is defined by [64]:

(50) $$erfc\left(x\right)=\frac{2}{\sqrt{\pi }}\int_{x}^{\infty }e^{{-z}^{2}}dz.$$

The derivation of the above formulas is given in Appendix A.4. The initial values $H_{k}\left(0\right)$ of Hermite polynomials are specified by [52]:

(51) $$H_{2k}\left(0\right)={\left(-1\right)}^{k}\left(2k\right)!/k!\;\mathrm{and}\;H_{2k+1}\left(0\right)=0.$$

**Figure 4:**
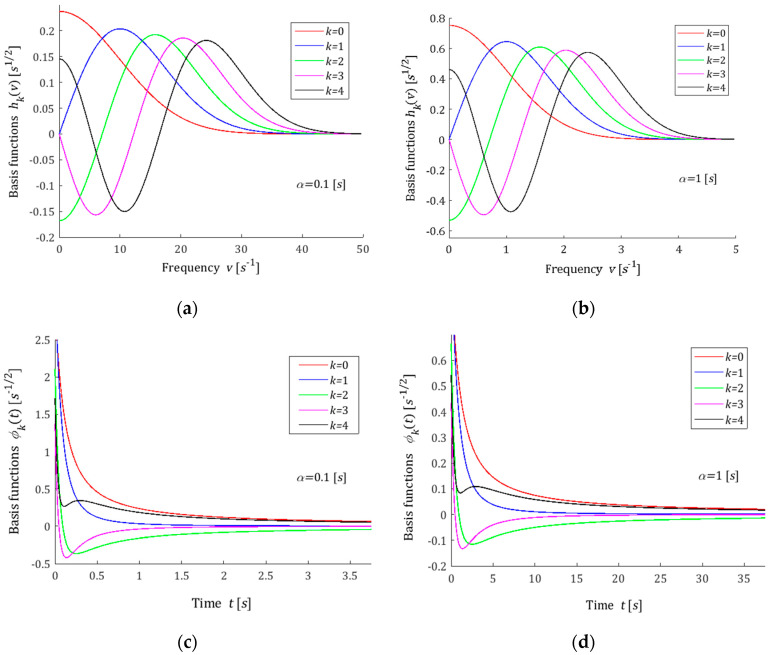
Basis functions $h_{k}\left(v\right)$ (44) and $\phi_{k}\left(t\right)$ (47)–(49) of the Hermite model for two time-scaling factors: (**a**) $h_{k}\left(v\right)$, α=0.1[s]; (**b**) $h_{k}\left(v\right)$, α=1[s]; (**c**) $\phi_{k}\left(t\right)$, α=0.1[s]; (**d**) $\phi_{k}\left(t\right)$, α=1[s]; k=0,1,2,3,4.

A few first basis functions $h_{k}\left(v\right)$ are shown in Figure 4a,b for two factors α. The related functions $\phi_{k}\left(t\right)$ are plotted in Figure 4c,d. The function $\phi_{0}\left(t\right)$, and, in consequence of the recursive Formula (47), all functions $\phi_{k}\left(t\right)$, depend on the exponential multiplier $e^{t^{2}/\left(2\alpha^{2}\right)}$ rapidly moving towards infinity. The following asymptotic properties were proved in Appendix A.5 for any α>0:

(52) $${\underset{t\to \infty }{lim}\;\phi }_{k}\left(t\right)=0,\;k=0,1,2,\ldots ,$$

(53) $${\underset{t\to \infty }{lim}\;t\phi }_{k}\left(t\right)=\frac{\alpha }{\sqrt{2^{k}\alpha k!}\sqrt[{4}]{\pi }}H_{k}\left(0\right)\;k=0,1,2,\ldots .$$

However, in numerical computations, the limited values of $\phi_{k}\left(t\right)$ can be guaranteed only for $t\leq t_{upp}$, where $t_{upp}$ depends on the maximal real number accessible in the computing environment. For example, in Matlab, the largest finite floating-point number in IEEE double precision $realmax=(2-2^{-52})\cdot 2^{1023}≅1.7977\cdot 10^{308}$. Thus, in view of (48), the range of numerical applicability of the Hermite model in the time domain, determined by the inequality

$$e^{t^{2}/\left(2\alpha^{2}\right)}\leq realmax,$$

is as follows

(54) $$t\leq t_{upp}=\sqrt{2\alpha^{2}\ln\left(realmax\right)}=\alpha \sqrt{2\ln\left(realmax\right)}≅37.6771\alpha .$$

#### Smoothness

Since the basis functions $h_{k}\left(v\right)$ of the Hermite model presented above form an orthonormal basis of the space $L^{2}\left(-\infty ,\right.\left.\infty \right)$ of square integrable functions on $\left(-\infty ,\right.\left.\infty \right)$, for Hermite model estimation (28) can be replaced by

$${\left\|{\overset{-}{H}}_{K}^{M}\left(v\right)\right\|}_{2}^{2}=\int_{0}^{\infty }{\left[{\overset{-}{H}}_{M}^{M}\left(v\right)\right]}^{2}dv\leq \int_{-\infty }^{\infty }{\left[{\overset{-}{H}}_{M}^{M}\left(v\right)\right]}^{2}dv=\sum_{k=0}^{K-1}{\left[{\overset{-}{g}}_{k}^{\lambda }\right]}^{2}={\left\|{\overset{-}{g}}_{K}^{\lambda }\right\|}_{2}^{2}.$$

Therefore, for the Hermite model, the algorithm may (but does not have to) provide a stronger limitation of the fluctuation in the determined relaxation spectrum ${\overset{-}{H}}_{K}^{M}\left(v\right)$ than for the other models. 

### 3.7. Choice of the Basis Functions

In the models proposed above, the parameter α>0 is the time-scaling factor. The following rule holds: the lower the parameter α is, the shorter the relaxation times are, i.e., the greater the relaxation frequencies are. The above is illustrated by Figure 1, Figure 2, Figure 3 and Figure 4. Through the optimal choice of the scaling factor, the best fit of the model to the experimental data can be achieved. However, in practice, a simple rough rule for choosing the factor α, based on the comparison of a few first functions from the sequence $\left\{\phi_{k}\left(t\right)\right\}$ for different values of α with the experimentally obtained function $\overset{-}{G}\left(t_{i}\right)$, is quite enough. In the same manner, the number K of the series $G_{K}\left(t\right)$ (8) elements can be initially evaluated. This rough selection strategy of the model parameters was used in the examples presented below. Thus, the choice of the number K and the parameter α must be carried out *a posteriori*, after the preliminary experiment data analysis. 

The ranges of applicability of the four classes of models described above in the relaxation times *t* domain and the relaxation frequencies v domain for different values of α are summarized in Table A1 in Appendix A.6. It was assumed that the range of applicability for times is determined by the value of time t, for which the first K=11 basis functions $\phi_{k}\left(t\right)$ no longer permanently exceed, i.e., for any θ>t, ε=0.5% of its maximum value. Specifically,

(55) $$t_{app}=\underset{0\leq k\leq K-1}{\max}\underset{t>0}{\min}\left\{t:\left|\phi_{k}\left(\theta \right)\right|\leq 0.005{\cdot \phi }_{kmax}\;for\;any\;\theta \geq t\right\},$$

where

$$\phi_{kmax}=\underset{t\geq 0}{\max}\left|\phi_{k}\left(t\right)\right|.$$

Similarly, the range of applicability for the relaxation frequencies was defined on the basis of the variability in the basis functions $h_{k}\left(v\right)$. Here,

(56) $$v_{app}=\underset{0\leq k\leq K-1}{\max}\underset{v>0}{\min}\left\{v:\left|h_{k}\left(\vartheta \right)\right|\leq 0.005\cdot h_{kmax}\;for\;any\;\vartheta \geq v\right\},$$

with $h_{kmax}$ defined by

$$h_{kmax}=\underset{v\geq 0}{\max}\left|h_{k}\left(v\right)\right|.$$

In view of the problems described above concerning the numerical determination of the basis functions only for $t\leq t_{upp}$, with $t_{upp}$ defined in (54), for the Hermite model, ε=0.0212 was assumed. 

### 3.8. Example 1

Consider viscoelastic material of relaxation spectrum described by Gauss-like distribution [11]

(57) $$H\left(v\right)=ve^{-{\left(v-2\right)}^{2}/3},$$

**Figure 5:**
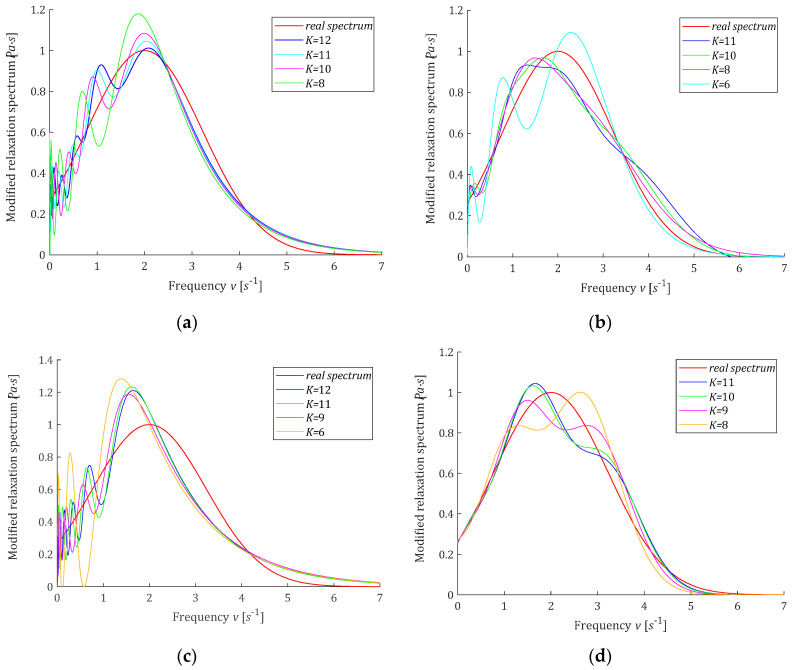
Modified relaxation spectrum $H^{M}\left(v\right)$(58) (solid red line) from Example 1 and the approximated models ${\overset{-}{H}}_{K}^{M}\left(v\right)$: (**a**) Legendre, (**b**) Laguerre, (**c**) Chebyshev, (**d**) Hermite.

**Figure 6:**
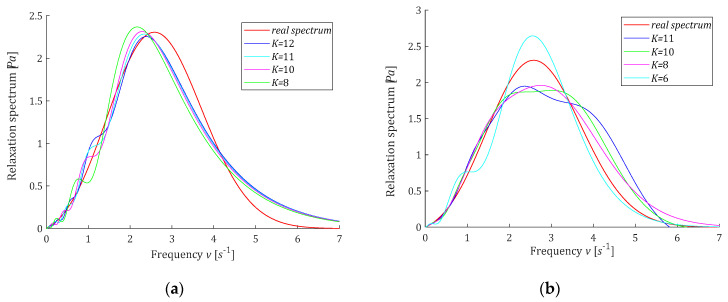

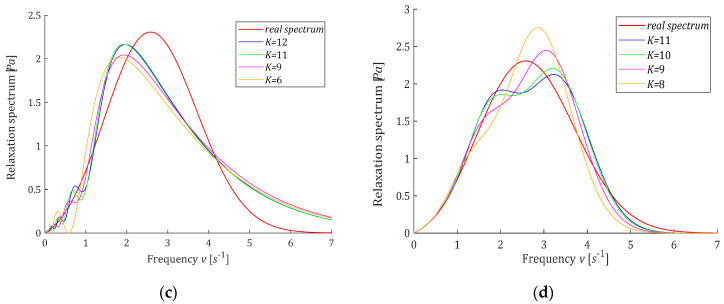
Relaxation spectrum $H\left(v\right)$ (57) (solid red line) from Example 1 and the approximated models ${\overset{-}{H}}_{K}\left(v\right)$ (23): (**a**) Legendre, (**b**) Laguerre, (**c**) Chebyshev, (**d**) Hermite.

**Figure A1:**
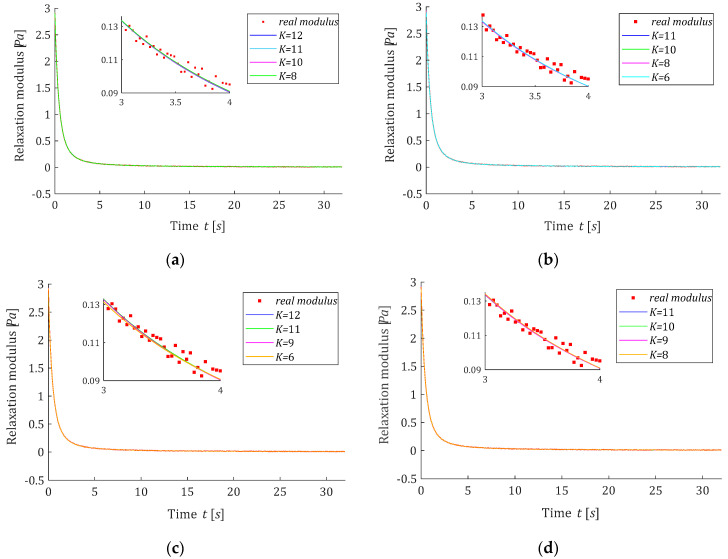
The measurements of the relaxation modulus $G\left(t\right)$ (59) (red points) from Example 1 and the approximated models $G_{K}\left(t\right)$ (8): (**a**) Legendre, (**b**) Laguerre, (**c**) Chebyshev, (**d**) Hermite.

The corresponding modified spectrum $H^{M}\left(v\right)$ (4) is:

(58) $$H^{M}\left(v\right)=\frac{H\left(v\right)}{v}=e^{-{\left(v-2\right)}^{2}/3}.$$

and, therefore, using (5), the ‘real’ relaxation modulus is

(59) $$G\left(t\right)=\int_{0}^{\infty }e^{-{\left(v-2\right)}^{2}/3}e^{-tv}dv=\frac{\sqrt{3\pi }}{2}e^{\frac{3}{4}t^{2}-2t}erfc\left(\frac{3t-4}{2\sqrt{3}}\right)$$

where erfc is defined by (50). In the experiment, N=1000 sampling instants $t_{i}$ were generated with the constant period in the time interval $T=\left[0,32\right]$ seconds selected in view of the course of the modulus $G\left(t\right)$ (59). Additive measurement noises $z\left(t_{i}\right)$ were selected independently by random choice with uniform distribution on the interval $\left[-0.005,0.005\right]Pa$, i.e., maximally 6.1% of the mean value of $G\left(t\right)$ in the interval T defined as the average value of the integral of $G\left(t\right)$ over T, which is equal to 0.0820Pa. The time-scaling factors α are selected by comparison for different values of α a few first functions $\phi_{k}\left(t\right)$ with the experiment results $\overset{-}{G}\left(t_{i}\right)$. Only for the Chebyshev model, the rough selection of α required several attempts; for the remaining classes of models, it was enough to review the data from Table A1. The basis functions $h_{k}\left(v\right)$ and $\phi_{k}\left(t\right)$ were simulated in Matlab R2022a using special functions *erfc*, *legendreP*, *chebyshevT*, and *hermiteH.* For the singular value decomposition procedure, *svd* was applied. For K=6,8,9,10,11, and sometimes also for K=12, the regularization parameters $\lambda_{GCV}$ were determined and are given in Table 1. Next, the vectors of optimal model parameters ${\overset{-}{g}}_{K}^{\lambda }={\overset{-}{g}}_{K}^{\lambda_{GCV}}$ (19) were computed and are given in Table A2 in Appendix B. Only for the Laguerre models, some elements of the vectors of optimal parameters are negative; for the remaining classes of models, all the parameter vectors ${\overset{-}{g}}_{K}^{\lambda_{GCV}}$ are positive. The optimal modified spectra of relaxation frequencies ${\overset{-}{H}}_{K}^{M}\left(v\right)$ (22) and the ‘real’ spectrum $H^{M}\left(v\right)$ (58) are plotted in Figure 5 for selected values of K, while in Figure 6, the spectra ${\overset{-}{H}}_{K}\left(v\right)$ (23) and $H\left(v\right)$ (57) are presented. The optimal models of the relaxation modulus $G_{K}\left(t\right)$ computed for $g_{K}={\overset{-}{g}}_{K}^{\lambda_{GCV}}$ according to (8) are plotted in Figure A1 in Appendix B, where the measurements $\overset{-}{G}\left(t_{i}\right)$ are also marked. Since the optimal models $G_{K}\left(t\right)$ (8) fitted the data extremely well, as indicated especially by the mean-square model errors $Q_{N}\left({\overset{-}{g}}_{K}^{\lambda_{GCV}}\right)/N$, which vary between $0.80\cdot 10^{-5}{Pa}^{2}$ and $0.117\cdot 10^{-4}{Pa}^{2}$, models $G_{K}\left(t\right)$ for different K practically coincide with the measurement points and with each other (see Figure A1). The norms ${\left\|{\overset{-}{g}}_{K}^{\lambda }\right\|}_{2}$ and ${\left\|{\overset{-}{H}}_{K}^{M}\left(v\right)\right\|}_{2}$, as the measures of the solution smoothness, and the identification index $Q_{N}\left({\overset{-}{g}}_{K}^{\lambda_{GCV}}\right)$ (10), being a measure of the errors of the relaxation modulus models, are also given in Table 1. For the ‘real’ modified spectrum $H^{M}\left(v\right)$ (58), the norm ${\left\|H^{M}\left(v\right)\right\|}_{2}=1.4656Pa\cdot s^{1/2}$. The distance between the ‘real’ spectrum $H^{M}\left(v\right)$ (58) and their models ${\overset{-}{H}}_{K}^{M}\left(v\right)$ (22) was estimated by integral square error, defined as:

(60) $${{ERR}^{2}=\left\|{\overset{-}{H}}_{K}^{M}\left(v\right)-H^{M}\left(v\right)\right\|}_{2}^{2}=\int_{0}^{\infty }{\left[{\overset{-}{H}}_{K}^{M}\left(v\right)-H^{M}\left(v\right)\right]}^{2}dv,$$

and is given in the last column of Table 1. 

### 3.9. Example 2 

**Figure 7:**
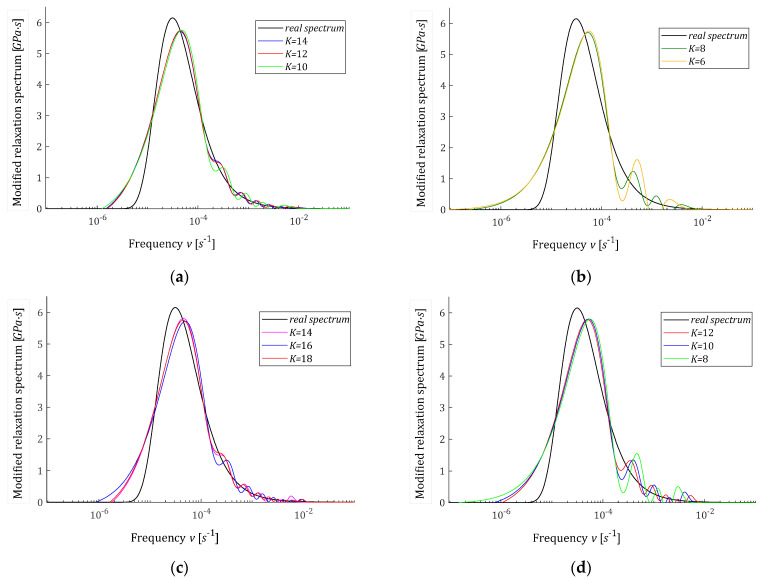
Modified relaxation spectrum $H^{M}\left(v\right)$ (61) (solid black line) from Example 2 and the approximated models ${\overset{-}{H}}_{K}^{M}\left(v\right)$: (**a**,**b**) Legendre models, (**c**,**d**) Laguerre models.

**Figure 8:**
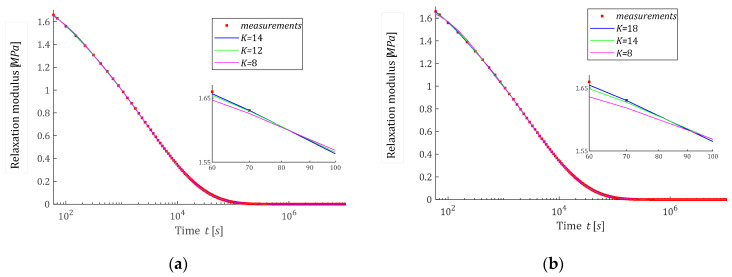

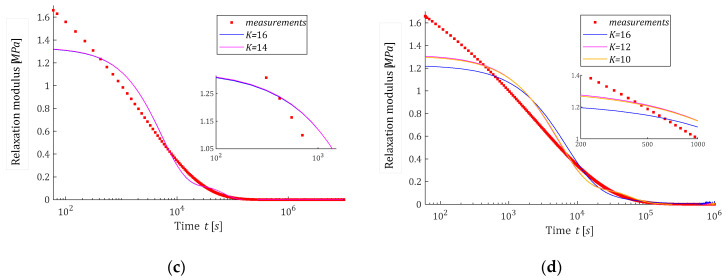
The measurements of the relaxation modulus $G\left(t\right)$ corresponding to the relaxation spectrum $H^{M}\left(v\right)$ (61) (red points) from Example 2 and the optimal approximated models $G_{K}\left(t\right)$ (8): (**a**) Legendre, (**b**) Laguerre, (**c**) Chebyshev, (**d**) Hermite.

Consider the spectrum of relaxation times introduced by Baumgaertel, Schausberger and Winter [36,37],

$$H\left(\tau \right)=\left\{\beta_{1}{\left(\frac{\tau }{\tau_{c}}\right)}^{\rho_{1}}+\beta_{2}{\left(\frac{\tau }{\tau_{c}}\right)}^{\rho_{2}}\right\}e^{-\frac{\tau }{\tau_{max}}},$$

which is known to be effective in describing polydisperse polymer melts [17,34], with the parameters [34]: $\beta_{1}=6.276\cdot 10^{4}Pa$, $\beta_{2}=1.27\cdot 10^{5}Pa$, $\tau_{c}=2.481s$, $\tau_{max}=2.564\cdot 10^{4}s$, $\rho_{1}=0.25$ and $\rho_{2}=-0.5$. The related spectra of relaxation frequencies $H\left(v\right)=H\left(\frac{1}{v}\right)$ and $H^{M}\left(v\right)$ (4) are well posed for v>0. The modified spectrum is described by

(61) $$H^{M}\left(v\right)=\frac{H\left(v\right)}{v}=\frac{1}{v}\left\{\beta_{1}{\left(\frac{1}{v\tau_{c}}\right)}^{\rho_{1}}+\beta_{2}{\left(\frac{1}{v\tau_{c}}\right)}^{\rho_{2}}\right\}e^{-\frac{1}{{v\tau }_{max}}}$$

and depicted in Figure 7; the corresponding ‘real’ relaxation modulus $G\left(t\right)$ is defined by (5). In the experiment, N=1000 time instants $t_{i}$ were sampled according to the square rule $t_{i}=∆t{\left(i-1\right)}^{2}+60$ s, with parameter $∆t=T/{\left(N-1\right)}^{2}$, where $T=10^{7}s$, in the time interval $T=\left[0,T\right]$ is selected in view of the course of the modulus $G\left(t\right)$. Due to the numerical problems described above related to determining the basis functions $\phi_{k}\left(t\right)$, Equations (47)–(49), for the Hermite model the experiment was simulated in a shorter time interval $T=10^{6}s$ using the same sampling formula. Additive measurement noises $z\left(t_{i}\right)$ were selected independently by random choice with uniform distribution on the interval $\left[-0.0005,0.0005\right]MPa$. Here, for the selection of parameter α, which guarantees a satisfactory accuracy of the modulus approximation, several or even a dozen or so attempts were necessary. This means that it will be advisable to further extend the algorithm by the level of optimal selection of the time-scaling factors. For K=6,8,10,12,14, and also for K=16, the regularization parameters $\lambda_{GCV}$ were determined and are given in Table 2. Only for the Laguerre and Legendre models, the accuracy of the modulus approximation measured by $Q_{N}\left({\overset{-}{g}}_{K}^{\lambda_{GCV}}\right)$, Equation (10), is comparable to that obtained in Example 1. Thus, the vectors of optimal model parameters ${\overset{-}{g}}_{K}^{\lambda }={\overset{-}{g}}_{K}^{\lambda_{GCV}}$ (19) for Legendre and Laguerre models are given in Table A3 in Appendix B; for the remaining models, they were omitted. The optimal spectra ${\overset{-}{H}}_{K}^{M}\left(v\right)$ (22) and the ‘real’ spectrum $H^{M}\left(v\right)$ (58) are plotted in Figure 7 for Laguerre and Legendre models; a logarithmic scale is used for the frequencies v and a linear scale for the spectrum. For Legendre and Laguerre models, the optimal models $G_{K}\left(t\right)$ computed for $g_{K}={\overset{-}{g}}_{K}^{\lambda_{GCV}}$ are plotted in Figure 8, where the measurements $\overset{-}{G}\left(t_{i}\right)$ are also marked and logarithmic scale is applied for time axis. The norms ${\left\|{\overset{-}{g}}_{K}^{\lambda }\right\|}_{2}$, ${\left\|{\overset{-}{H}}_{K}^{M}\left(v\right)\right\|}_{2}$ and the identification index $Q_{N}\left({\overset{-}{g}}_{K}^{\lambda_{GCV}}\right)$ are given in Table 2. For the ‘real’ modified spectrum $H^{M}\left(v\right)$ (58), the norm ${\left\|H^{M}\left(v\right)\right\|}_{2}=56.9809MPa\cdot s^{1/2}$. The distance between the ‘real’ spectrum $H^{M}\left(v\right)$ (58) and its models ${\overset{-}{H}}_{K}^{M}\left(v\right)$ (22) was estimated by the integral square errors ERR, defined in (60) and given in Table 2, only for Legendre and Laguerre models. For the Chebyshev and Hermite models, a satisfactory quality of approximation was not obtained, despite the research carried out for a wide range of α values. The best found α and related identification indices $Q_{N}\left({\overset{-}{g}}_{K}^{\lambda_{GCV}}\right)$ are given in Table 2, but other indices are omitted. The optimal Legendre and Laguerre models $G_{K}\left(t\right)$ (8) were well fitted to the experimental data, see Figure 8a,b, as also indicated by the mean-square model errors $Q_{N}\left({\overset{-}{g}}_{K}^{\lambda_{GCV}}\right)/N$, which vary in a range from $5.27\cdot 10^{-7}{MPa}^{2}$ to $3.26\cdot 10^{-6}{MPa}^{2}$. For the Chebyshev and Hermite models, even increasing the number of model summands K does not improve the poor approximation of the relaxation modulus data (see Figure 8c,d and the values of $Q_{N}\left({\overset{-}{g}}_{K}^{\lambda_{GCV}}\right)$ from Table 2). In particular, the course of the $G_{K}\left(t\right)$ model from Figure 8c shows that it is not too few components of the model but the properties of the Chebyshev model that make it ineffective for approximating the relaxation spectrum $H^{M}\left(v\right)$ (61).

### 3.10. Remarks

The example orthogonal basis functions for relaxation spectrum models have been assumed as the products of exponentials and Legendre, Laguerre, Chebyshev and Hermite polynomials. For Legendre and Laguerre models, the basis functions $\phi_{k}\left(t\right)$ of the relaxation modulus are rational functions; for the Chebyshev model, they are determined by the quotient of the Euler’s gamma functions, which is a generalization of the factorial of a non-negative integer for the no-integer argument, while, for the Hermite model, these functions are based on the complementary error function. From Figure 1c,d and Figure 2c,d, it is evident that the basis functions $\phi_{k}\left(t\right)$ for the relaxation modulus of Legendre and Laguerre models are in good agreement with the real relaxation modulus obtained in the experiment. However, Figure 3c,d and Figure 4c,d show that, for the Chebyshev and Hermite models, so good agreement is not achieved for individual functions $\phi_{k}\left(t\right)$. Hence, a significantly worse fit of the Chebyshev and Hermite models to the measurement data for the spectrum from Example 2, which has a much wider range of relaxation frequencies, results.

Both examples show that, generally, increasing the number of model summands improves the model quality, provided that the assumed series can provide a good approximation of the relaxation modulus. If a given series of special functions is not suitable for approximation of the relaxation modulus for a given material, then, as shown by the research conducted for the Chebyshev and Hermite models in the second example, increasing the number of series components does not improve the quality of the model.

## 4. Conclusions

In this paper, a class of algorithms for the relaxation spectrum identification, which combines the technique of an expansion of a function into a series in an orthonormal basis with the least-squares regularized identification, was derived. It was demonstrated that due to the choice of an appropriate special functions (Legendre, Laguerre, Chebyshev, Hermite) as the basis functions for the relaxation spectrum model, the basis functions of the relaxation modulus model are given by compact analytical or recursive formulas. Due to the choice of orthonormal basis, the smoothness of the vector of the optimal model parameters implies equivalent smoothness of the fluctuations in the model of the relaxation spectrum.

The proposed approach based on the expansion of the relaxation spectrum into a function series can be applied for arbitrary basis functions. The proven convergence and noise robustness properties of the optimal models will be retained, but the smoothing of the spectrum model will require separate analysis. As part of further research, the algorithm can be extended with a superior level of optimal selection of the time-scaling factor, so as to obtain a better fit to the measurement data. The presented scheme of the relaxation spectrum identification can be easily modified for the retardation spectrum recovery from the creep compliance measurements obtained in the standard creep test.

## Figures and Tables

**Table 1:** The parameters of the optimal models in Example 1: time-scale factors α, numbers of model summands K, regularization parameter $\lambda_{GCV}$ and the approximation error indices: identification index $Q_{N}\left({\overset{-}{g}}_{K}^{\lambda_{GCV}}\right)$, Equation (10), the errors ERR (60) of the relaxation spectrum models.

Model	K	α [s]	$\lambda_{GCV}$ [-]	$Q_{N}\left({\overset{-}{g}}_{K}^{\lambda_{GCV}}\right)$ $\left[{Pa}^{2}\right]$	${\left\|{\overset{-}{g}}_{K}^{\lambda }\right\|}_{2}$ $\left[Pa\cdot s^{1/2}\right]$	${\left\|{\overset{-}{H}}_{K}^{M}\left(v\right)\right\|}_{2}$ $\left[Pa\cdot s^{1/2}\right]$	ERR $\left[Pa\cdot s^{1/2}\right]$
Legendre model	6	1	0.03378	0.0088	1.5518	1.5518	2.0782
	8	1	0.03239	0.0083	1.4833	1.4833	2.0643
	9	1	0.03186	0.0084	1.4657	1.4657	2.0597
	10	1	0.03139	0.0087	1.4537	1.4537	2.0559
	11	1	0.03098	0.0089	1.4452	1.4452	2.0529
	12	1	0.03062	0.0091	1.4391	1.4391	2.0505
Laguerre model	6	6	0.03259	0.0084	1.4929	1.4929	1.9914
	8	6	0.02744	0.0093	1.4222	1.4222	2.0414
	9	6	0.02560	0.0092	1.4240	1.4240	2.0435
	10	6	0.0239	0.0089	1.4262	1.4262	2.0448
	11	6	0.02256	0.0088	1.4253	1.4253	2.0444
Chebyshev model	6	1.5	0.03103	0.00992	1.5215	1.5215	2.1119
	8	1.5	0.03077	0.00981	1.4677	1.4677	2.0811
	9	1.5	0.03062	0.0101	1.4543	1.4543	2.0668
	10	1.6	0.03165	0.0089	1.4875	1.4875	2.0864
	11	1.6	0.03148	0.0091	1.47492	1.4749	2.0775
	12	1.6	0.03126	0.009246	1.4651	1.4651	2.0713
Hermite model	6	1	0.00625	0.0117	1.6405	1.6029	2.0529
	8	1	0.00588	0.0081	1.5077	1.4891	2.0674
	9	1	0.00599	0.0080	1.4731	1.4639	2.0592
	10	1	0.00599	0.0080	1.4611	1.4569	2.0607
	11	1	0.00566	0.0080	1.4600	1.4561	2.0654

**Table 2:** The parameters of the optimal models in Example 2: time-scale factors α, numbers of model summands K, regularization parameter $\lambda_{GCV}$, optimal model parameters ${\overset{-}{g}}_{K}^{\lambda_{GCV}}$, approximation error indices: identification index $Q_{N}\left({\overset{-}{g}}_{K}^{\lambda_{GCV}}\right)$, Equation (10), the errors ERR (60) of the relaxation spectrum models.

Model	K	α [s]	$\lambda_{GCV}$ [-]	$Q_{N}\left({\overset{-}{g}}_{K}^{\lambda_{GCV}}\right)$ $\left[{MPa}^{2}\right]$	${\left\|{\overset{-}{g}}_{K}^{\lambda }\right\|}_{2}$ $\left[MPa\cdot s^{1/2}\right]$	${\left\|{\overset{-}{H}}_{K}^{M}\left(v\right)\right\|}_{2}$ $\left[MPa\cdot s^{1/2}\right]$	ERR $\left[MPa\cdot s^{1/2}\right]$
Legendre model	6	950	2.72 × 10^−6^	0.003261	62.717	62.717	80.604
	8	620	4.968 × 10^−6^	0.00135	58.9351	58.9351	79.7444
	10	500	6.372 × 10^−6^	0.0007138	57.4422	57.4422	79.7170
	12	400	7.754 × 10^−6^	0.000557	56.8251	56.8251	79.7917
	14	300	9.226 × 10^−6^	0.000575	56.6390	56.6390	79.8353
Laguerre model	8	8500	2.524 × 10^−6^	0.002722	63.0250	63.0250	82.4674
	10	8000	3.668 × 10^−6^	0.001494	59.7092	59.7092	81.0828
	12	7500	4.840 × 10^−6^	0.0009379	58.3032	58.3032	80.6344
	14	8000	5.294 × 10^−6^	0.000711	57.3277	57.3277	80.3069
	16	6000	7.498 × 10^−6^	0.000699	57.39886	57.39886	80.4441
	18	6500	7.686 × 10^−6^	0.000527	56.7967	56.7967	80.1877
Chebyshev model	6	31,000	6.0 × 10^−7^	0.8259	289.32	289.32	
	8	31,500	6.0 × 10^−7^	0.7115	346.30	346.30	
	10	32,000	5.0 × 10^−7^	0.6504	376.6564	376.66	
	12	33,000	5.0 × 10^−7^	0.6344	379.0402	379.04	
	14	33,000	5.0 × 10^−7^	0.6029	375.3072	375.31	
	16	34,500	5.0 × 10^−7^	0.6136	382.0129	382.01	
Hermite model	6	27,000	1.8 × 10^−6^	3.0971	390.24	387.13	
	8	26,800	1.6 × 10^−6^	2.1911	452.31	445.67	
	10	26,750	1.6 × 10^−6^	1.9010	440.42	436.32	
	12	26,700	1.8 × 10^−6^	1.8148	389.93	378.76	
	14	26,600	0.999 × 10^−6^	2.9235	472.33	467.32	
	16	26,600	0.994 × 10^−6^	2.7767	467.45	461.56	

## Data Availability

Not applicable.

## Nomenclature

$G\left(t\right)$	linear relaxation modulus, Equation (1), Pa
$G\left(t_{i}\right)$	relaxation modulus obtained in stress relaxation test, i=1,…,N , Pa
$\overset{-}{G}\left(t_{i}\right)$	noise corrupted $\mathrm{measurement}\;\mathrm{of}\;\mathrm{the}\;\mathrm{modulus}\;G\left(t_{i}\right)$, i=1,…,N, Pa
$G_{K}\left(t\right)$	relaxation modulus model corresponding to $H_{K}^{M}\left(v\right)$, Equation (8), Pa
${\overset{-}{G}}_{N}$	N dimensional vector of the measurements $\overset{-}{G}\left(t_{i}\right)$, Equation (11)
$G_{N}$	N dimensional vector of noise-free measurements $G\left(t_{i}\right)$
$g_{k}$	parameters of the $\mathrm{model}\;H_{K}^{M}\left(v\right)$, k=0,1,…K−1,$\;\mathrm{Equation}\;(7),\;Pa\cdot s^{1/2}$
$g_{K}$	$\mathrm{vector}\;\mathrm{of}\;\mathrm{parameters}\;g_{k}$$\;\mathrm{of}\;\mathrm{the}\;\mathrm{models}\;H_{K}^{M}\left(v\right)$$\;(7)\;\mathrm{and}\;G_{K}\left(t\right)$ (8)
${\overset{-}{g}}_{K}^{\lambda }$	solution of regularized task (14) given for regularization parameter λ by (15) or (19)
${\tilde{g}}_{K}^{\lambda }$	solution of regularized task (14) for ideal measurements, Equation (25)
${\overset{-}{g}}_{K}^{\lambda_{GCV}}$	vector of optimal model parameters determined by GCV method
${\overset{-}{g}}_{K}^{N}$ $,\;{\tilde{g}}_{K}^{N}$	normal solutions of least-squares problem (13) for noise and noise-free measurements of the relaxation modulus
$H\left(v\right)$	continuous spectrum of relaxation frequencies v, Equation (3), Pa
$H^{M}\left(v\right)$	modified relaxation spectrum, Equation (4), Pa·s
$H_{K}^{M}\left(v\right)$	$\mathrm{model}\;\mathrm{of}\;\mathrm{the}\;\mathrm{modified}\;\mathrm{relaxation}\;\mathrm{spectrum}\;H^{M}\left(v\right)$ , Equation (7), Pa·s
${\overset{-}{H}}_{K}^{M}\left(v\right)$	model of $\mathrm{modified}\;\mathrm{spectrum}\;H^{M}\left(v\right)$$\;\mathrm{for}\;‘\mathrm{regularized}’\;\mathrm{parameter}\;{\overset{-}{g}}_{K}^{\lambda }$, Equation (22), Pa·s
${\overset{-}{H}}_{K}\left(v\right)$	model of $\mathrm{spectrum}\;H\left(v\right)$$\;\mathrm{for}\;‘\mathrm{regularized}’\;\mathrm{parameter}\;{\overset{-}{g}}_{K}^{\lambda }$, Equation (23), Pa
${\tilde{H}}_{K}^{M}\left(v\right)$	model of $H^{M}\left(v\right)$ obtained for ideal measurements, Equation (24), Pa·s
${\overset{-}{H}}_{K}^{N}\left(v\right)$ $,\;{\tilde{H}}_{K}^{N}\left(v\right)$	models of $H^{M}\left(v\right)$ obtained for the normal solutions ${\overset{-}{g}}_{K}^{N}$$,\;{\tilde{g}}_{K}^{N}$ described by Equations (26) and (27), Pa
$h_{k}\left(v\right)$	basis functions of the $\mathrm{model}\;H_{K}^{M}\left(v\right)$, k=0,1,…K−1, Equation (7), Pa·s
$I_{K,K}$	K dimensional identity matrix
K	number of $\mathrm{model}\;H_{K}^{M}\left(v\right)$ summands, Equation (7), –
N	number of measurements in the stress relaxation test, –
$Q_{N}\left(g_{K}\right)$	square identification index defined by (10), ${Pa}^{2}$
r	number of non-zero singular values of $\Phi_{N,K}$, –
$t_{i}$	sampling instants used in the stress relaxation test, i=1,…,N , s
T	time interval from which the sampling instants $t_{i}$ were generated
V , U	orthogonal matrices of singular value decomposition of $\Phi_{N,K}$, Equation (18)
$V_{GCV}\left(\lambda \right)$	the GCV functional defined by (16) and expressed by (21)
Y	N dimensional vector $Y=U^{T}{\overset{-}{G}}_{N}$
$z\left(t_{i}\right)$	additive noise of relaxation modulus measurement, i=1,…,N , Pa
$z_{N}$	N dimensional vector $\mathrm{of}\;\mathrm{measurement}\;\mathrm{noises}\;z\left(t_{i}\right)$, i=1,…,N, Pa
α	time-scaling factor of the basis functions $h_{k}\left(v\right)$, s
λ	regularization parameter introduced in the optimization task (14), –
$\lambda_{GCV}$	optimal regularization parameter determined by GCV method, Equation (17)
$\Lambda_{\lambda }$	K×N diagonal matrix defined by (20)
$\sigma_{i}$	the non-zero singular values of $\Phi_{N,K}$, i=1,2,…,r, Equation (18)
Σ	N×K diagonal matrix of singular values $\sigma_{1},\ldots ,\sigma_{r}$ of $\Phi_{N,K}$, Equation (18)
$\Phi_{N,K}$	N×K matrix defined by (11) basis for least-squares task (13)
$\Phi_{N,K}^{†}$	$\mathrm{the}\;\mathrm{Moore}-\mathrm{Penrose}\;\mathrm{pseudoinverse}\;\mathrm{of}\;\mathrm{matrix}\;\Phi_{N,K}$
$\phi_{k}\left(t\right)$	$\mathrm{basis}\;\mathrm{functions}\;\mathrm{defined}\;\mathrm{by}\;(9)\;\mathrm{of}\;\mathrm{the}\;\mathrm{model}\;G_{K}\left(t\right)$ $,\;\mathrm{Equation}\;(8),\;s^{-1/2}$
ERR	integral square error of $H^{M}\left(v\right)$ approximation, Equation (60), $Pa\cdot s^{1/2}$

## Appendix A

### Appendix A.1. Derivation of the Formula (32)

Mathematical induction will be used. In the proof, the following recurrence representation of the Legendre polynomials [52,53]:

(A1) $$P_{k}\left(x\right)=\frac{2k-1}{k}xP_{k-1}\left(x\right)-\frac{k-1}{k}P_{k-2}\left(x\right),k=2,3,\ldots ,$$

is used, where the two first polynomials are as follows

(A2) $$P_{0}\left(x\right)=1,$$

and

(A3) $$P_{1}\left(x\right)=x.$$

In the base case of the proof by induction, for k=0 and k=1, Formula (32) follows immediately from definition (9) and properties (A2), (A3). For k=0, by (9) (31) and (A2) the basis function $\phi_{k}\left(t\right)$:

$$\phi_{0}\left(t\right)=\int_{0}^{\infty }\sqrt{2\alpha }e^{-\alpha v}e^{-tv}dv=\sqrt{2\alpha }\frac{1}{t+\alpha }=\frac{\sqrt{2\alpha }}{\prod_{i=0}^{0}\left[\left(2i+1\right)\alpha +t\right]},$$

while for k=1, applying (A3), we obtain:

$$\phi_{1}\left(t\right)=\frac{\sqrt{6\alpha }}{t+\alpha }-\frac{2\sqrt{6\alpha }}{t+3\alpha }=\sqrt{2\alpha \left(2+1\right)}\frac{\prod_{i=0}^{0}\left[\left(2i+1\right)\alpha -t\right]}{\prod_{i=0}^{1}\left[\left(2i+1\right)\alpha +t\right]}.$$

For k≥2, in the induction step, combining definitions (9) and (31), we have:

(A4) $$\phi_{k}\left(t\right)=\int_{0}^{\infty }\sqrt{2\alpha \left(2k+1\right)}e^{-\alpha v}P_{k}\left(1-2e^{-2\alpha v}\right)e^{-tv}dv,$$

where, substituting $P_{k}\left(x\right)$ given by recurrence Equation (A1) and having in mind (A4), we obtain:

$$\phi_{k}\left(t\right)=\frac{2k-1}{k}\frac{\sqrt{2\alpha \left(2k+1\right)}}{\sqrt{2\alpha \left(2k-1\right)}}\phi_{k-1}\left(t\right)-\frac{2\left(2k-1\right)}{k}\frac{\sqrt{2\alpha \left(2k+1\right)}}{\sqrt{2\alpha \left(2k-1\right)}}\phi_{k-1}\left(t+2\alpha \right)-\;\frac{k-1}{k}\frac{\sqrt{2\alpha \left(2k+1\right)}}{\sqrt{2\alpha \left(2k-3\right)}}\phi_{k-2}\left(t\right).$$

Via the induction hypothesis, $\phi_{k-2}\left(t\right)$ and $\phi_{k-1}\left(t\right)$ are given by (32); thus, the basis function $\phi_{k}\left(t\right)$ can be rewritten as:

(A5) $$\phi_{k}\left(t\right)=\;\frac{\sqrt{2\alpha \left(2k+1\right)}}{k}\left[\left(2k-1\right)\frac{\prod_{i=0}^{k-2}\left[\left(2i+1\right)\alpha -t\right]}{\prod_{i=0}^{k-1}\left[\left(2i+1\right)\alpha +t\right]}-2\left(2k-1\right)\frac{\prod_{i=0}^{k-2}\left[\left(2i+1\right)\alpha -t-2\alpha \right]}{\prod_{i=0}^{k-1}\left[\left(2i+1\right)\alpha +t+2\alpha \right]}-\;\left(k-1\right)\frac{\prod_{i=0}^{k-3}\left[\left(2i+1\right)\alpha -t\right]}{\prod_{i=0}^{k-2}\left[\left(2i+1\right)\alpha +t\right]}\right].$$

Since

$$\frac{\prod_{i=0}^{k-2}\left[\left(2i+1\right)\alpha -t-2\alpha \right]}{\prod_{i=0}^{k-1}\left[\left(2i+1\right)\alpha +t+2\alpha \right]}=\frac{\left(-1\right)\left(t+\alpha \right)\prod_{i=0}^{k-3}\left[\left(2i+1\right)\alpha -t\right]}{\prod_{i=1}^{k}\left[\left(2i+1\right)\alpha +t\right]},$$

function $\phi_{k}\left(t\right)$ (A5) is given by

$$\phi_{k}\left(t\right)=\;\frac{\sqrt{2\alpha \left(2k+1\right)}}{k}\left[\left(2k-1\right)\frac{\prod_{i=0}^{k-2}\left[\left(2i+1\right)\alpha -t\right]}{\prod_{i=0}^{k-1}\left[\left(2i+1\right)\alpha +t\right]}+2\left(2k-1\right)\frac{\left(t+\alpha \right)\prod_{i=0}^{k-3}\left[\left(2i+1\right)\alpha -t\right]}{\prod_{i=1}^{k}\left[\left(2i+1\right)\alpha +t\right]}-\;\left(k-1\right)\frac{\prod_{i=0}^{k-3}\left[\left(2i+1\right)\alpha -t\right]}{\prod_{i=0}^{k-2}\left[\left(2i+1\right)\alpha +t\right]}\right],$$

and, after algebraic manipulations, can be expressed as:

$$\phi_{k}\left(t\right)=\sqrt{2\alpha \left(2k+1\right)}\frac{\prod_{i=0}^{k-2}\left[\left(2i+1\right)\alpha -t\right]}{\prod_{i=0}^{k-1}\left[\left(2i+1\right)\alpha +t\right]}\frac{\left[\left(2k-1\right)\alpha -t\right]}{\left[\left(2k+1\right)\alpha +t\right]}.$$

Hence, the direct Formula (32) follows immediately.

### Appendix A.2. Derivation of Formula (35)

To derive the basis function $\phi_{k}\left(t\right)$ (35), we start with the alternative formula of the Laguerre polynomials $L_{k}\left(v\right)$ (33) given by [65]:

(A6) Lkv=∑j=0kkk−j−αvjj!, k=0,1,2,…

An easy consequence of (A6) is

hkv=α∑j=0kkk−j−αjj!vje−αv/2,k=0,1,2,…

where, using the well-known formula for the Laplace transform

$$L\left[v^{j}e^{-\alpha v/2}\right]=\frac{j!}{{\left(t+\frac{\alpha }{2}\right)}^{j+1}},\;j=0,1,2,\ldots ,$$

the basis functions $\phi_{k}\left(t\right)$ defined by (9) are given by

ϕkt=α∑j=0kkk−j−αjt+α2j+1

for k=0,1,2,…. Hence, by binomial theorem, we have

ϕkt=αt+α2k+1∑j=0kkk−j−αjt+α2k−j=αt+α2k+1t−α2k,

where Formula (35) follows. This formula is known in [55] and coincides with that given in [66].

### Appendix A.3. Derivation of Formulas (40)–(43)

Suppose α>0. Let us first derive Formulas (41), (42). For k=0 by (39) and (9), we have

(A7) $$\phi_{0}\left(t\right)=\int_{0}^{\infty }h_{0}\left(v\right)e^{-tv}dv=\sqrt{\frac{2\alpha }{\pi }}\int_{0}^{\infty }{\left(e^{2\alpha v}-1\right)}^{-1/4}e^{-tv}dv,$$

which, after substitution $e^{-2\alpha v}=u$, is equivalent to

$$\phi_{0}\left(t\right)=\frac{1}{\sqrt{2\alpha \pi }}\int_{0}^{1}{\left(\frac{1}{u}-1\right)}^{-\frac{1}{4}}u^{\frac{t}{2\alpha }-1}du=\frac{1}{\sqrt{2\alpha \pi }}\int_{0}^{1}{\left(1-u\right)}^{-\frac{1}{4}}u^{\frac{t}{2\alpha }-\frac{3}{4}}du,$$

whereby, due to the well-known Euler–Poisson integral [60]:

$$\int_{0}^{1}{\left(1-u\right)}^{n-1}u^{m-1}du=\frac{\Gamma \left(n\right)\Gamma \left(m\right)}{\Gamma \left(n+m\right)},$$

we immediately obtain (41).

For k=1, by (38) and (9) for the polynomial $T_{1}\left(x\right)=x$, the integral $\phi_{1}\left(t\right)$ is given by

(A8) $$\phi_{1}\left(t\right)=2\sqrt{\frac{\alpha }{\pi }}\int_{0}^{\infty }{\left(e^{2\alpha v}-1\right)}^{-1/4}{\left(1-2e^{-2\alpha v}\right)e}^{-tv}dv,$$

and, keeping in mind the last expression in (A7), can be rewritten as

$$\phi_{1}\left(t\right)=\sqrt{2}\phi_{0}\left(t\right)-2\sqrt{2}\phi_{0}\left(t+2\alpha \right).$$

Then, from Equation (41), we obtain

(A9) $$\phi_{1}\left(t\right)=\frac{1}{\sqrt{\pi \alpha }}\frac{\Gamma \left(\frac{3}{4}\right)\Gamma \left(\frac{t}{2\alpha }+\frac{1}{4}\right)}{\Gamma \left(\frac{t}{2\alpha }+1\right)}-2\frac{1}{\sqrt{\pi \alpha }}\frac{\Gamma \left(\frac{3}{4}\right)\Gamma \left(\frac{t+2\alpha }{2\alpha }+\frac{1}{4}\right)}{\Gamma \left(\frac{t+2\alpha }{2\alpha }+1\right)}=\frac{\Gamma \left(\frac{3}{4}\right)}{\sqrt{\pi \alpha }}\left[\frac{\Gamma \left(\frac{t}{2\alpha }+\frac{1}{4}\right)}{\Gamma \left(\frac{t}{2\alpha }+1\right)}-2\frac{\Gamma \left(\frac{t}{2\alpha }+\frac{1}{4}+1\right)}{\Gamma \left(\frac{t}{2\alpha }+2\right)}\right],$$

Using the well-known property of the Gamma function [60]:

(A10) $$\Gamma \left(x+1\right)=x\Gamma \left(x\right),$$

Equation (A9), after algebraic manipulations, can be expressed as

(A11) $$\phi_{1}\left(t\right)=\frac{\left(\alpha -t\right)}{\sqrt{\pi \alpha }\left(t+2\alpha \right)}\frac{\Gamma \left(\frac{3}{4}\right)\Gamma \left(\frac{t}{2\alpha }+\frac{1}{4}\right)}{\Gamma \left(\frac{t}{2\alpha }+1\right)},$$

or in equivalent form as Formula (42).

For k=2, by (36), (37) and (9), we have

$$\phi_{2}\left(t\right)=2\sqrt{\frac{\alpha }{\pi }}\int_{0}^{\infty }{\left(e^{2\alpha v}-1\right)}^{-1/4}\left[2\left(1-2e^{-2\alpha v}\right)T_{1}\left(1-2e^{-2\alpha v}\right)-1\right]e^{-tv}dv,$$

which, keeping in mind (A8) and (A7), can be rewritten as follows

$$\phi_{2}\left(t\right)=2\phi_{1}\left(t\right)-4\phi_{1}\left(t+2\alpha \right)-\sqrt{2}\phi_{0}\left(t\right),$$

and where, by (41), (A11) and (A10), after algebraic manipulations, we obtain

(A12) $$\phi_{2}\left(t\right)=\frac{\left(2\alpha^{2}+t^{2}-6\alpha t\right)}{\sqrt{\pi \alpha }\left(t+2\alpha \right)\left(t+4\alpha \right)}\frac{\Gamma \left(\frac{3}{4}\right)\Gamma \left(\frac{t}{2\alpha }+\frac{1}{4}\right)}{\Gamma \left(\frac{t}{2\alpha }+1\right)},$$

or, equivalently, Equation (43).

Now, it remains to show that (40) holds for any k≥3. Recalling the identity (36) and using (38) for k≥3, we have

$$h_{k}\left(v\right)=2\left(1-2e^{-2\alpha v}\right)h_{k-1}\left(v\right)-h_{k-2}\left(v\right)=2h_{k-1}\left(v\right)-h_{k-2}\left(v\right)-4e^{-2\alpha v}h_{k-1}\left(v\right)$$

where, bearing in mind definition (9), we obtain (40), which concludes the proof.

### Appendix A.4. Derivation of Formulas (47)–(49)

Let us first derive the formulas (48) and (49). Since $H_{0}\left(x\right)=1$, for k=0, by applying in the integral $\phi_{0}\left(t\right)$ the substitution $u=\left(\alpha v+t/\alpha \right)/\sqrt{2}$, we have:

(A13) $$\phi_{0}\left(t\right)=\frac{\sqrt{\alpha }}{\sqrt[{4}]{\pi }}\int_{0}^{\infty }e^{-{\left(\alpha v\right)}^{2}/2}e^{-tv}dv=\frac{\sqrt{2}}{\sqrt{\alpha }\sqrt[{4}]{\pi }}e^{t^{2}/\left(2\alpha^{2}\right)}\int_{t/\left(\sqrt{2}\alpha \right)}^{\infty }e^{{-u}^{2}}du,$$

where, by definition (50) of the complementary error function, Formula (48) is immediately obtained.

For k=1, the polynomial $H_{1}\left(\alpha v\right)=2\alpha v$; then, the integral $\phi_{1}\left(t\right)$ is as follows

$$\phi_{1}\left(t\right)=\frac{\sqrt{2\alpha }\alpha }{\sqrt[{4}]{\pi }}\int_{0}^{\infty }ve^{-{\left(\alpha v\right)}^{2}/2}e^{-tv}dv.$$

Comparing the first integral in Formula (A13) and the integral $\phi_{1}\left(t\right)$, it is easy to notice that

(A14) $$\phi_{1}\left(t\right)=-\sqrt{2}\alpha \phi_{0}^{\prime }\left(t\right).$$

Since by (50), we have

(A15) $$\frac{derfc\left(t/\left(\sqrt{2}\alpha \right)\right)}{dt}=-\frac{\sqrt{2}}{\alpha \sqrt{\pi }}e^{{-t}^{2}/\left(2\alpha^{2}\right)},$$

Equations (A14) and (48) yield

$$\phi_{1}\left(t\right)=-\frac{\sqrt[{4}]{\pi }}{\sqrt{\alpha }}\left[\frac{t}{\alpha }e^{t^{2}/\left(2\alpha^{2}\right)}erfc\left(\frac{t}{\sqrt{2}\alpha }\right)-\frac{\sqrt{2}}{\sqrt{\pi }}e^{t^{2}/\left(2\alpha^{2}\right)}e^{{-t}^{2}/\left(2\alpha^{2}\right)}\right],$$

where the next formula follows

(A16) $$\phi_{1}\left(t\right)=\frac{\sqrt[{4}]{\pi }}{\sqrt{\alpha }}\left[\frac{\sqrt{2}}{\sqrt{\pi }}-\frac{t}{\alpha }e^{t^{2}/\left(2\alpha^{2}\right)}erfc\left(\frac{t}{\sqrt{2}\alpha }\right)\right],$$

and, in view of (48), Formula (49) results.

The proof of the recursive Equation (47) is based on (45), which, by definitions (44) and (9) for k≥2, yields

(A17) $$\phi_{k}\left(t\right)=\frac{2\alpha \sqrt{\alpha }}{\sqrt{2^{k}k!}\sqrt[{4}]{\pi }}\int_{0}^{\infty }ve^{-{\left(\alpha v\right)}^{2}/2}H_{k-1}\left(\alpha v\right)e^{-tv}dv-\;\frac{2\left(k-1\right)\sqrt{\alpha }}{\sqrt{2^{k}k!}\sqrt[{4}]{\pi }}\int_{0}^{\infty }e^{-{\left(\alpha v\right)}^{2}/2}H_{k-2}\left(\alpha v\right)e^{-tv}dv.$$

By the integration by parts, the first integral in (A17) is as follows

$$I_{k}\left(t\right)=\int_{0}^{\infty }ve^{-{\left(\alpha v\right)}^{2}/2}H_{k-1}\left(\alpha v\right)e^{-tv}dv={\left.-\frac{1}{\alpha^{2}}e^{-{\left(\alpha v\right)}^{2}/2}H_{k-1}\left(\alpha v\right)e^{-tv}\right|}_{0}^{\infty }+\;\frac{1}{\alpha }\int_{0}^{\infty }e^{-{\left(\alpha v\right)}^{2}/2}{H^{\prime }}_{k-1}\left(\alpha v\right)e^{-tv}dv-\frac{t}{\alpha^{2}}\int_{0}^{\infty }e^{-{\left(\alpha v\right)}^{2}/2}H_{k-1}\left(\alpha v\right)e^{-tv}dv,$$

which, bearing in mind that

$$\underset{v\to \infty }{\lim}\left[e^{-{\left(\alpha v\right)}^{2}/2}H_{k-1}\left(\alpha v\right)e^{-tv}\right]=0,$$

and taking into account the known property of Hermite polynomials [52]:

$$H_{k}^{\prime }\left(x\right)=2kH_{k-1}\left(x\right)$$

and definitional Formulas (44), (9), can be expressed as

$$I_{k}\left(t\right)=\frac{1}{\alpha^{2}}H_{k-1}\left(0\right)+\frac{2\left(k-1\right)\sqrt{2^{k-2}\left(k-2\right)!}\sqrt[{4}]{\pi }}{\alpha \sqrt{\alpha }}\phi_{k-2}\left(t\right)-\frac{\sqrt{2^{k-1}\left(k-1\right)!}\sqrt[{4}]{\pi }}{\alpha^{2}\sqrt{\alpha }}{t\phi }_{k-1}\left(t\right),$$

which, combined with (A17), finally yields

$$\phi_{k}\left(t\right)=\;\frac{2\alpha \sqrt{\alpha }}{\sqrt{2^{k}k!}\sqrt[{4}]{\pi }}\left[\frac{1}{\alpha^{2}}H_{k-1}\left(0\right)+\frac{2\left(k-1\right)\sqrt{2^{k-2}\left(k-2\right)!}\sqrt[{4}]{\pi }}{\alpha \sqrt{\alpha }}\phi_{k-2}\left(t\right)-\;\frac{\sqrt{2^{k-1}\left(k-1\right)!}\sqrt[{4}]{\pi }}{\alpha^{2}\sqrt{\alpha }}{t\phi }_{k-1}\left(t\right)\right]-\frac{2\left(k-1\right)\sqrt{\alpha }}{\sqrt{2^{k}k!}\sqrt[{4}]{\pi }}\frac{\sqrt{2^{k-2}\left(k-2\right)!}\sqrt[{4}]{\pi }}{\sqrt{\alpha }}\phi_{k-2}\left(t\right),$$

where, after algebraic manipulations, Formula (47) follows.

### Appendix A.5. Proof of Formulas (52) and (53)

Mathematical induction will be used again. In the base step, we prove (52) and (53), successively, for k=0, k=1 and k=2. By (48), we have

(A18) $$\phi_{0}\left(t\right)=\frac{\sqrt[{4}]{\pi }}{\sqrt{2\alpha }}e^{t^{2}/\left(2\alpha^{2}\right)}erfc\left(\frac{t}{\sqrt{2}\alpha }\right)=\frac{\sqrt[{4}]{\pi }}{\sqrt{2\alpha }}\frac{erfc\left(t/\left(\sqrt{2}\alpha \right)\right)}{e^{-t^{2}/\left(2\alpha^{2}\right)}},$$

where both the numerator and denominator of the expression on the right hand side of (A18) become zero when t→∞. Thus, using the L’Hospital’s rule, and taking into account (A15), we obtain

$$\underset{t\to \infty }{lim\;}\phi_{0}\left(t\right)=\frac{\sqrt[{4}]{\pi }}{\sqrt{2\alpha }}\underset{t\to \infty }{lim}\frac{-\frac{\sqrt{2}}{\alpha \sqrt{\pi }}e^{{-t}^{2}/\left(2\alpha^{2}\right)}}{-\frac{t}{\alpha^{2}}e^{-t^{2}/\left(2\alpha^{2}\right)}}=\frac{\sqrt[{4}]{\pi }}{\sqrt{2\alpha }}\underset{t\to \infty }{lim}\frac{\sqrt{2}\alpha }{\sqrt{\pi }t}=0.$$

Analogically,

(A19) $$\underset{t\to \infty }{lim}t\phi_{0}\left(t\right)=\frac{\sqrt[{4}]{\pi }}{\sqrt{2\alpha }}\underset{t\to \infty }{lim}\frac{erfc\left(t/\left(\sqrt{2}\alpha \right)\right)}{{\frac{1}{t}e}^{-t^{2}/\left(2\alpha^{2}\right)}}=\frac{\sqrt[{4}]{\pi }}{\sqrt{2\alpha }}\underset{t\to \infty }{lim}\frac{\frac{\sqrt{2}}{\alpha \sqrt{\pi }}}{\frac{1}{\alpha^{2}}+\frac{1}{t^{2}}}=\frac{\sqrt[{4}]{\pi }}{\sqrt{2\alpha }}\frac{\sqrt{2}}{\sqrt{\pi }}\alpha =\frac{\sqrt{\alpha }}{\sqrt[{4}]{\pi }},$$

where, since by (51) the initial condition $H_{0}\left(0\right)=1$, we have

$$\underset{t\to \infty }{lim}\;t\varphi_{0}\left(t\right)=\frac{\sqrt{\alpha }}{\sqrt[{4}]{\pi }}H_{0}\left(0\right).$$

Now, by (A19) and (49), we have

$$\underset{t\to \infty }{lim\;}\phi_{1}\left(t\right)=\frac{\sqrt{2}}{\sqrt{\alpha }\sqrt[{4}]{\pi }}-\frac{\sqrt{2}}{\alpha }\frac{\sqrt{\alpha }}{\sqrt[{4}]{\pi }}=0.$$

In order to prove (53) for k=1, it is enough to prove that $t\phi_{1}\left(t\right)\to 0$, when t→∞, since by (51), the initial condition $H_{1}\left(0\right)=0$. On the basis of (A16), we have

(A20) $$\underset{t\to \infty }{lim\;}t\phi_{1}\left(t\right)=\frac{\sqrt[{4}]{\pi }}{\sqrt{\alpha }}\underset{t\to \infty }{lim}\frac{\left[\frac{\sqrt{2}}{\sqrt{\pi }}{\frac{1}{t}e}^{{-t}^{2}/\left(2\alpha^{2}\right)}-\frac{1}{\alpha }erfc\left(t/\left(\sqrt{2}\alpha \right)\right)\right]}{\frac{e^{{-t}^{2}/\left(2\alpha^{2}\right)}}{t^{2}}}.$$

Thus, using the L’Hospital’s rule and keeping in mind (A15), we obtain

$$\underset{t\to \infty }{lim}t\phi_{1}\left(t\right)=\frac{\sqrt{2}}{\sqrt{\alpha }\sqrt[{4}]{\pi }}\underset{t\to \infty }{lim}\frac{1}{\frac{1}{\alpha^{2}}t+\frac{2}{t}}=0,$$

where, since based on (47) and (51), we have

(A21) $$\phi_{2}\left(t\right)=\sqrt{\frac{1}{2}}\phi_{0}\left(t\right)-\frac{1}{\alpha }{t\phi }_{1}\left(t\right),$$

we immediately obtain $\underset{t\to \infty }{lim\;}\phi_{2}\left(t\right)=0$. By (A21) and (A19), the limit

(A22) $$\underset{t\to \infty }{lim\;}t\phi_{2}\left(t\right)=\underset{t\to \infty }{lim}\left[\sqrt{\frac{1}{2}}{t\phi }_{0}\left(t\right)-\frac{1}{\alpha }{t^{2}\phi }_{1}\left(t\right)\right]=\frac{\sqrt{\alpha }}{\sqrt{2}\sqrt[{4}]{\pi }}-\frac{1}{\alpha }\underset{t\to \infty }{lim}{t^{2}\phi }_{1}\left(t\right).$$

Based on (A16)

$$\underset{t\to \infty }{lim\;}{t^{2}\phi }_{1}\left(t\right)=\frac{\sqrt[{4}]{\pi }}{\sqrt{\alpha }}\underset{t\to \infty }{lim}\frac{\left[\frac{\sqrt{2}}{\sqrt{\pi }}{\frac{1}{t}e}^{{-t}^{2}/\left(2\alpha^{2}\right)}-\frac{1}{\alpha }erfc\left(\frac{t}{\sqrt{2}\alpha }\right)\right]}{\frac{e^{{-t}^{2}/\left(2\alpha^{2}\right)}}{t^{3}}}.$$

Hence, similarly as in the case of (A20), using the L’Hospital’s rule and including Formula (A15), after simple transformations, we obtain

(A23) $$\underset{t\to \infty }{lim\;}{t^{2}\phi }_{1}\left(t\right)=\frac{\sqrt[{4}]{\pi }}{\sqrt{\alpha }}\underset{t\to \infty }{lim}\frac{-\frac{\sqrt{2}}{\sqrt{\pi }t^{2}}}{\frac{\frac{-1}{\alpha^{2}}t^{4}-3t^{2}}{t^{6}}}=\frac{\sqrt[{4}]{\pi }}{\sqrt{\alpha }}\underset{t\to \infty }{lim}\frac{-\frac{\sqrt{2}}{\sqrt{\pi }}}{\frac{-1}{\alpha^{2}}-\frac{3}{t^{2}}}=\frac{\sqrt[{4}]{\pi }}{\sqrt{\alpha }}\frac{\frac{\sqrt{2}}{\sqrt{\pi }}}{\frac{1}{\alpha^{2}}}=\frac{\sqrt[{4}]{\pi }}{\sqrt{\alpha }}\frac{\frac{\sqrt{2}}{\sqrt{\pi }}}{\frac{1}{\alpha^{2}}}=\frac{\alpha^{2}\sqrt{2}}{\sqrt{\alpha }\sqrt[{4}]{\pi }}.$$

Next, substituting (A23) into (A22) yields

$$\underset{t\to \infty }{lim\;}t\phi_{2}\left(t\right)=\frac{\sqrt{\alpha }}{\sqrt{2}\sqrt[{4}]{\pi }}-\frac{1}{\alpha }\frac{\alpha^{2}\sqrt{2}}{\sqrt{\alpha }\sqrt[{4}]{\pi }}=\frac{-\alpha }{\sqrt{2}\sqrt{\alpha }\sqrt[{4}]{\pi }}=\frac{\left(-2\right)\alpha }{\sqrt{2^{2}\alpha 2!}\sqrt[{4}]{\pi }},$$

and bearing in mind that by (51) $H_{2}\left(0\right)=-2$, Equation (53) is proved for k=2.

Now, in the induction step, let us assume that Formulas (52) and (53) hold for k−1, k−2 and k−3, where k≥3. We prove that they hold also for k. Consider the recursive Formula (47). By the induction hypothesis, including (52) for k−2 and (53) for k−1, from (47), we immediately have

$${\underset{t\to \infty }{lim\;}\phi }_{k}\left(t\right)=\frac{1}{\sqrt{2^{k-2}k!\alpha }\sqrt[{4}]{\pi }}H_{k-1}\left(0\right)-\frac{\sqrt{2}}{\sqrt{k}\alpha }\frac{\alpha }{\sqrt{2^{k-1}\alpha \left(k-1\right)!}\sqrt[{4}]{\pi }}H_{k-1}\left(0\right)=0.$$

It is only necessary to prove the correctness of Formula (53) for k. The limit ${\underset{t\to \infty }{lim}t\phi }_{k}\left(t\right)$ can be rewritten equivalently as follows

(A24) $${\underset{t\to \infty }{lim\;}t\phi }_{k}\left(t\right)=\underset{t\to \infty }{lim}\frac{\frac{1}{t}e^{{-t}^{2}/\left(2\alpha^{2}\right)}\phi_{k}\left(t\right)}{\frac{e^{{-t}^{2}/\left(2\alpha^{2}\right)}}{t^{2}}},$$

where both the numerator and denominator of the expression on the right expression of (A24) become zero, when t→∞. Using the L’Hospital’s rule, we have

(A25) $${\underset{t\to \infty }{lim\;}t\phi }_{k}\left(t\right)=\underset{t\to \infty }{lim}\frac{\left[\frac{1}{t}+\frac{1}{\alpha^{2}}t\right]\phi_{k}\left(t\right)-\phi_{k}^{\prime }\left(t\right)}{\frac{1}{\alpha^{2}}+\frac{2}{t^{2}}}.$$

On the basis of (47):

(A26) $$\phi_{k}^{\prime }\left(t\right)=\sqrt{\frac{k-1}{k}}\phi_{k-2}^{\prime }\left(t\right)-\frac{\sqrt{2}}{\sqrt{k}\alpha }\phi_{k-1}\left(t\right)-\frac{\sqrt{2}}{\sqrt{k}\alpha }t\phi_{k-1}^{\prime }\left(t\right)$$

To determine the derivative $\phi_{k-2}^{\prime }\left(t\right)$, note that based on (9) and (44), we have

(A27) $$\phi_{k-2}^{\prime }\left(t\right)=-\frac{\sqrt{\alpha }}{\sqrt{2^{k-2}\left(k-2\right)!}\sqrt[{4}]{\pi }}\int_{0}^{\infty }ve^{-{\left(\alpha v\right)}^{2}/2}H_{k-2}\left(\alpha v\right)e^{-tv}dv.$$

Since by (45)

(A28) $$H_{k-1}\left(\alpha v\right)=2\alpha vH_{k-2}\left(\alpha v\right)-2\left(k-2\right)H_{k-3}\left(\alpha v\right)$$

Equation (A27) can be expreseed as

$$\phi_{k-2}^{\prime }\left(t\right)=\;-\frac{\sqrt{\alpha }}{2\alpha \sqrt{2^{k-2}\left(k-2\right)!}\sqrt[{4}]{\pi }}\left[\int_{0}^{\infty }e^{-{\left(\alpha v\right)}^{2}/2}H_{k-1}\left(\alpha v\right)e^{-tv}dv+2\left(k-\;2\right)\int_{0}^{\infty }e^{-{\left(\alpha v\right)}^{2}/2}H_{k-3}\left(\alpha v\right)e^{-tv}dv\right]$$

where, in view of (44) and (9), after algebraic manipulations, the next formula results

(A29) $$\phi_{k-2}^{\prime }\left(t\right)=-\left[\frac{\sqrt{k-1}}{\sqrt{2}\alpha }\phi_{k-1}\left(t\right)+\frac{\sqrt{k-2}}{\sqrt{2}\alpha }\phi_{k-3}\left(t\right)\right].$$

Similarly, for k−1, we have

(A30) $$\phi_{k-1}^{\prime }\left(t\right)=-\left[\frac{\sqrt{k}}{\sqrt{2}\alpha }\phi_{k}\left(t\right)+\frac{\sqrt{k-1}}{\sqrt{2}\alpha }\phi_{k-2}\left(t\right)\right].$$

Now, combining (A26), (A30) and (A29), after algebraic manipulations, yields

(A31) $$\phi_{k}^{\prime }\left(t\right)=-\frac{k-1}{\sqrt{2k}\alpha }\phi_{k-1}\left(t\right)-\frac{\sqrt{\left(k-1\right)\left(k-2\right)}}{\sqrt{2k}\alpha }\phi_{k-3}\left(t\right)-\frac{\sqrt{2}}{\sqrt{k}\alpha }\phi_{k-1}\left(t\right)+\;\frac{1}{\alpha^{2}}t\phi_{k}\left(t\right)+\frac{\sqrt{k-1}}{\sqrt{k}\alpha^{2}}t\phi_{k-2}\left(t\right).$$

Substituting the derivative $\phi_{k}^{\prime }\left(t\right)$ (A31) into (A25), and bearing in mind that $\phi_{k-3}\left(t\right)$, $\phi_{k-1}\left(t\right)$ and $\phi_{k}\left(t\right)$ tend to zero as t→∞, we obtain

$${\underset{t\to \infty }{lim}t\phi }_{k}\left(t\right)=\underset{t\to \infty }{lim}\frac{-\frac{\sqrt{k-1}}{\sqrt{k}\alpha^{2}}t\phi_{k-2}\left(t\right)}{\frac{1}{\alpha^{2}}+\frac{2}{t^{2}}}.$$

By induction hypothesis, (53) holds for $\left(k-2\right)$. Thus, we have

(A32) $${\underset{t\to \infty }{lim\;}t\phi }_{k}\left(t\right)=\frac{-\frac{\sqrt{k-1}}{\sqrt{k}\alpha^{2}}}{\frac{1}{\alpha^{2}}}\frac{\alpha }{\sqrt{2^{k-2}\alpha \left(k-2\right)!}\sqrt[{4}]{\pi }}H_{k-2}\left(0\right)=-\frac{\alpha \left(k-1\right)}{\sqrt{2^{k-2}\alpha k!}\sqrt[{4}]{\pi }}H_{k-2}\left(0\right),$$

where, since by (45), the initial conditions are related by $H_{k}\left(0\right)=-2\left(k-1\right)H_{k-2}\left(0\right)$, the correctness of Formula (53) for k immediately follows from (A32).

### Appendix A.6. Applicability of the Models for Various Time-Scale Parameters

**Table A1:** Ranges of the applicability of the models for various time-scale parameters.

Model	Time-Scale Factor α [s]	Range ^1^ of Relaxation Frequency v $\left[s^{-1}\right]$	Range ^1^ of Time t [s]
Legendre model	0.001	5301	3.615
	0.01	530	36.15
	0.1	53	361.5
	1	5.3	3614.6
	10	0.53	36,144.5
	100	0.05301	361,445
	1000	0.005301	3,614,450
	10,000	0.0005301	36,144,500
Laguerre model	0.01	5256	0.995
	0.1	525.36	9.95
	1	52.5359	99.5
	10	5.2536	995
	100	0.528	9950
	1000	0.0526	99,500
	10,000	0.00526	995,000
	100,000	0.000526	9,950,000
Chebyshev model	0.001	10,020	0.34125
	0.01	1002	3.4125
	0.1	100.2	34.125
	1	10.02	341.25
	10	1.002	3412.5
	100	0.1002	34,125
	1000	0.01002	341,250
	10,000	0.001002	3,412,500
	100,000	0.0001002	34,125,000
Hermite model	0.001	4797.5	0.037639
	0.01	479.75	0.376394
	0.1	47.975	3.7639
	1	4.7975	37.63944
	10	0.47975	376.3944
	100	0.047975	3763.944
	1000	0.0047975	37,639.443
	10,000	0.00047975	376,394.436
	100,000	0.000047975	3,763,944.359

^1^ Only the upper bounds $t_{app}$ (55) and $v_{app}$ (56) of the respective intervals $\left[0,t_{app}\right]$ and $\left[0,v_{app}\right]$ are given.

## Appendix B

**Table A2:** Optimal parameters ${\overset{-}{g}}_{K}^{\lambda_{GCV}}$ of the relaxation spectrum models from Example 1; the elements of the vectors ${\overset{-}{g}}_{K}^{\lambda_{GCV}}$ are expressed in $\left[Pa\cdot s^{1/2}\right]$.

Model	${\overset{-}{g}}_{K}^{\lambda_{GCV}}$					
	K=6	K=8	K=9	K=10	K=11	K=12
Legendre model	0.8222	0.8225	0.8229	0.8232	0.8235	0.8238
	0.7699	0.7695	0.7705	0.7714	0.7723	0.7731
	0.6138	0.5979	0.5963	0.5959	0.5963	0.5970
	0.5299	0.4503	0.4347	0.4263	0.4215	0.4188
	0.5448	0.3564	0.3215	0.3021	0.2889	0.2801
	0.4300	0.3190	0.2887	0.2647	0.2490	0.2378
		0.2807	0.2442	0.2227	0.2074	0.1966
		0.2520	0.2185	0.1945	0.1781	0.1663
			0.1973	0.1742	0.1578	0.1462
				0.1562	0.1398	0.1280
					0.1264	0.1146
						0.1030
Laguerre model	0.3277	0.3265	0.3265	0.3266	0.3267	
	−0.5817	−0.5814	−0.5804	−0.5795	−0.5805	
	0.7416	0.7908	0.7897	0.7891	0.7856	
	−0.6558	−0.7164	−0.7193	−0.7163	−0.7109	
	0.7138	0.5043	0.5076	0.5013	0.5015	
	−0.5418	−0.36570	−0.3658	−0.3777	−0.3717	
		0.2063	0.2083	0.2121	0.2282	
		−0.0935	−0.0918	−0.1178	−0.1254	
			−0.0054	0.0122	0.0446	
				0.0517	0.0269	
					−0.0712	
Chebyshev model	0.8227	0.8265	0.8316	0.7988	0.8023	0.7991
	0.8350	0.8226	0.8139	0.7962	0.7905	0.7944
	0.5313	0.5312	0.5382	0.5234	0.5287	0.5248
	0.5168	0.4545	0.4362	0.4502	0.4397	0.4416
	0.4625	0.3392	0.3233	0.3549	0.3426	0.3294
	0.4209	0.2912	0.2623	0.3195	0.2992	0.2828
		0.2444	0.2138	0.2768	0.2542	0.2403
		0.2148	0.1899	0.2509	0.2317	0.2146
			0.1603	0.2270	0.2047	0.1899
				0.2112	0.1917	0.1752
					0.1734	0.1585
						0.1487
Hermite model	0.5504	0.6391	0.5909	0.6044	0.6051	
	0.7337	0.7670	0.8046	0.8004	0.8009	
	0.7965	0.6140	0.6703	0.6826	0.6853	
	0.6881	0.5241	0.4749	0.5005	0.5075	
	0.4916	0.5137	0.4089	0.3846	0.3885	
	0.7075	0.4046	0.3715	0.2971	0.2875	
		0.2833	0.2769	0.2366	0.2181	
		0.3419	0.2239	0.2318	0.2207	
			0.2576	0.2218	0.2178	
				0.1315	0.1381	
					0.0236	

**Table A3:** Optimal parameters ${\overset{-}{g}}_{K}^{\lambda_{GCV}}$ of the relaxation spectrum models from Example 2; the elements of the vectors ${\overset{-}{g}}_{K}^{\lambda_{GCV}}$ are expressed in $\left[MPa\cdot s^{1/2}\right]$.

**Model**	${\overset{-}{g}}_{K}^{\lambda_{GCV}}$					
	K=6	K=8	K=10	K=12	K=14	K=16
Legendre model	43.80739	39.6666	37.4618	35.1566	32.2127	
	−29.51378	−31.6618	−32.1025	−32.1325	−31.6435	
	8.10418	15.9237	19.1145	21.6308	23.8515	
	−2.20279	−6.1369	−8.1839	−10.9840	−14.3753	
	−3.819709	0.0198	1.5667	3.9792	7.5618	
	32.529089	10.4212	5.8515	2.9019	−0.9375	
		−10.2725	−5.9794	−4.2456	−1.7853	
		19.8011	11.2957	8.1554	5.3793	
			−8.6168	−6.9530	−5.7798	
			12.6105	9.3899	7.7029	
				−7.0433	−6.9429	
				8.7841	7.9943	
					−6.6946	
					7.3213	
	K=8	K=10	K=12	K=14	K=16	K=18
Laguerre model	53.0965	53.0609	53.0093	53.0664	52.5153	52.7585
	−1.4762	0.2502	1.8283	0.4988	6.3911	4.8727
	6.9102	6.5809	6.4712	5.2459	8.0061	6.8685
	−0.2900	−3.7059	−4.5349	−6.4194	−2.4884	−4.0192
	−10.8544	−6.2248	−3.8522	−3.7269	−0.8334	−1.5307
	−10.7846	−6.6303	−6.0659	−6.4034	−4.9840	−5.6574
	−8.9629	−8.7827	−7.0219	−6.1421	−4.2199	−4.2360
	−28.0790	−12.2858	−8.2334	−6.5918	−6.1999	−6.0076
		−6.1049	−6.7165	−5.7225	−5.4599	−4.9298
		−18.5624	−10.7187	−7.3355	−6.9589	−6.0759
			−4.8687	−4.0801	−5.6896	−4.7692
			−13.0786	−8.3498	−7.4589	−6.0712
				−2.0153	−5.4359	−4.2447
				−9.4015	−7.7715	−6.0352
					−4.9662	−3.5890
					−7.9368	−5.9731
						−2.9159
						−5.8840

## Appendix C

### Mathematical Terminology and Special Functions

$erfc\left(x\right)$	complementary error function, Equation (50)
$H_{k}\left(x\right)$	Hermite polynomials, k=0,1,2,…, Equations (45) and (46)
$L_{k}\left(x\right)$	Laguerre polynomials, k=0,1,2,…, Equation (33)
$P_{k}\left(x\right)$	Legendre polynomials, k=0,1,2,…, Equations (A1)–(A3)
$T_{k}\left(x\right)$	Chebyshev polynomials of the first kind, k=0,1,2,…, Equations (36) and (37)
$L^{2}\left(0,\right.\left.\infty \right)$	space of real-valued square-integrable functions on the interval $\left(0,\right.\left.\infty \right)$
$L\left[f\left(v\right)\right]$	Laplace transform of the function $f\left(v\right)$
$\Gamma \left(n\right)$	Euler’s gamma function
${\left\|\cdot \right\|}_{2}$	square norm in the real Euclidean space $R^{N}$ and in $L^{2}\left(0,\right.\left.\infty \right)$
$\underset{x}{min}f\left(x\right)$	find the value of x, which minimizes the function $f\left(x\right)$
